# The Swiss Personalized Health Network Metadata Catalog: Platform for Health Data Discovery and Exploration Based on Findable, Accessible, Interoperable, and Reusable Principles

**DOI:** 10.2196/90146

**Published:** 2026-07-14

**Authors:** Harald Witte, Deepak Raveendran Unni, Philip Krauss, Vasundra Touré, Jan Armida, Sabine Österle

**Affiliations:** 1 Swiss Personalized Health Network, SIB Swiss Institute of Bioinformatics, Elisabethenstrasse 43Basel, 4051, Switzerland, 41 765395411; 2 Accenture (Switzerland), Basel, Switzerland

**Keywords:** SPHN, metadata catalog, DCAT, HealthDCAT, schema visualization, data visualization, FAIR Data Point, W3C semantic standards, semantic web

## Abstract

**Background:**

The Swiss Personalized Health Network (SPHN) facilitates the interoperability and secure sharing of health-related data for research in Switzerland, in line with the findable, accessible, interoperable, and reusable (FAIR) principles. Since medical datasets can be highly sensitive, access is often governed by complex legal and regulatory requirements. Enabling researchers to discover, understand, and evaluate datasets through rich, well-structured metadata is therefore essential to support informed decisions about data suitability and reuse.

**Objective:**

This study describes the design and functionality of the SPHN Metadata Catalog and its role in supporting the discovery, exploration, and reuse assessment of health-related datasets.

**Methods:**

The SPHN Metadata Catalog is a FAIR Data Point–compliant infrastructure that provides rich, structured metadata in both human and machine-readable form. Dataset descriptions are based on the HealthDCAT (Health Data Catalog Vocabulary) Application Profile, ensuring a standardized representation of health data catalogs. Beyond the descriptive metadata typically offered by other catalogs, the SPHN Metadata Catalog includes extensive dataset-level statistics expressed using the Vocabulary of Interlinked Datasets. An interactive visualization component further enables users to explore graph-based schemas and datasets, including entities, attributes, relationships, and their relative abundances.

**Results:**

The SPHN Metadata Catalog enables users to explore the semantic structure of graph schemas and statistics of datasets prior to requesting access. Researchers can examine data structures, relationships, attributes, and the abundances of individual data elements. This functionality supports feasibility assessments and informed evaluations of dataset suitability and reuse conditions.

**Conclusions:**

By combining HealthDCAT Application Profile–based descriptions with rich statistical metadata and interactive exploration capabilities, the SPHN Metadata Catalog enhances dataset discoverability and supports FAIR-compliant data reuse. As a key component of Switzerland’s health data research infrastructure, the SPHN Metadata Catalog provides a foundation for future interoperability initiatives, including potential alignment with emerging frameworks such as the European Health Data Space.

## Introduction

### Health-Related Datasets: Challenges and Opportunities

With the rapid advances in digitalization and data-driven biomedical research, the volume and complexity of health-related datasets have grown significantly in recent years. These datasets, such as those within the Swiss Personalized Health Network (SPHN) [1], are often collected across multiple institutions and hold immense potential for advancing personalized medicine and clinical decision-making. Preparing these datasets for research use requires substantial time and effort for the curation, standardization, and cleaning of data.

Despite the effort to generate them, curated datasets are rarely reused outside their original consortia. One seemingly trivial reason is unawareness about suitable datasets due to their limited discoverability. Another reason is the legal and regulatory access restrictions to clinical datasets. Such datasets often comprise sensitive patient-related data and therefore cannot simply be shared with or previewed to third parties potentially interested in reuse. The provision of rich, findable, and easily accessible metadata, that is, data about data, would be a workaround to these key barriers by improving insights into the contents of a dataset without revealing sensitive information. Unfortunately, structured and accessible metadata that adequately describes the dataset content is often lacking. In many cases, available metadata is limited to high-level descriptive information, which provides insufficient insight into the structure, semantics, and actual content of the datasets. As a result, researchers frequently struggle to identify relevant datasets or, due to incomplete metadata, are unable to make a well-informed decision if the dataset is useful for their specific research questions. This ultimately hinders the broader reuse of these valuable data resources.

### Findable, Accessible, Interoperable, and Reusable Data Principles and Metadata Standards

The Findable, Accessible, Interoperable, and Reusable (FAIR) data principles, formally introduced in 2016 [2], establish guidelines to ensure that data can be discoverable, accessible, and reusable by both humans and machines. While these principles have gained widespread adoption, the FAIR principles build on a lineage of initiatives aimed at improving metadata standards and data stewardship. Foundational efforts such as the Dublin Core Metadata Initiative (DCMI) [3], the Data Documentation Initiative (DDI) [4] in the social sciences, and the Open Archival Information System reference model (International Organization for Standardization 14721:2003) [5] established essential models for structuring metadata and long-term preservation. The Semantic Web vision [6] further advanced the idea of machine-readable, linked metadata to enhance data integration and discoverability.

Several well-established and widely adopted standards from the World Wide Web Consortium (W3C) [7] provide a standardized vocabulary to describe digital objects:

- DCMI Metadata Terms [8] provides a foundation for expressing common metadata elements that describe resources. For example, “title” (dcterms:title) can be used to record the name of a resource, while “description” (dcterms:description) provides a free-text summary of its contents. The “publisher” (dcterms:publisher) property specifies the organization responsible for publishing a resource, and “license” (dcterms:license) links to the license governing how the resource may be reused. Additional properties such as “issued” (dcterms:issued) and “modified” (dcterms:modified) capture the date of publication and date of modification, respectively, ensuring that users and machines can clearly interpret the resource’s lifecycle.
- Data Catalog Vocabulary (DCAT) [9] builds upon DCMI Metadata Terms and is specifically designed for describing datasets, where a “Catalog” (dcat:Catalog) represents a curated collection of one or more datasets. A “Dataset” (dcat:Dataset) describes the data itself along with relevant metadata, and a “Distribution” (dcat:Distribution) represents a specific way the dataset is made available, either as a downloadable file or via an API endpoint. To improve discoverability, catalogs and datasets are tagged, represented via “keyword” (dcat:keyword), and can be categorized using one or more themes, represented via “theme” (dcat:theme).
- DCAT Application Profile for data portals in Europe (DCAT-AP) [10] is a specification built on DCAT that supports the description of public sector datasets across Europe. Its semantically interoperable design enables cross-portal searches, making public data easier to find and access across different countries and domains.
- The DCAT Application Profile for data portals in Switzerland (DCAT-AP CH) [11] is a specialized subprofile of the European DCAT-AP using a national extension of the DCAT vocabulary. Developed by the Swiss community, it provides clear guidance for data publishers in Switzerland on how to structure and describe their data catalogs and datasets in alignment with the European standard. This guarantees semantic interoperability, allowing Swiss datasets to be discovered and reused not only within Switzerland but also across European data portals.
- FAIR Data Point Ontology (FDP-O) [12] builds on top of DCAT and provides the formal structure of metadata that is required for compatibility with the FAIR Data Point (FDP) implementation.
- Vocabulary of Interlinked Datasets (VoID) [13] is designed to describe metadata about Resource Description Framework (RDF) datasets, serving as a bridge between data publishers and consumers. It supports applications such as cataloging and describing datasets. For example, “entities” (void:entities) can be used to represent how many instances exist in the data, whereas “triples” (void:triples) can be used to represent how many triples an RDF dataset holds.

In addition to these general ones, there are also metadata standards for describing health-related datasets:

- HealthDCAT Application Profile (HealthDCAT-AP) [14] extends DCAT with health-specific metadata elements. It enables the capture of information relevant to biomedical and clinical datasets, further strengthening the discoverability and findability of biomedical data assets. For example, the property “population coverage” (healthdcatap:populationCoverage) describes the population from which the data were derived; details on access modalities for potentially sensitive datasets can be expressed via “access rights” (dcat:accessRights), while “hdab” (healthdcatap:hdab) records the health data access body that is responsible for governing access to the dataset.
- Health-RI metadata schema [15] has been developed under the Health Research Infrastructure (Health-RI) initiative [16] in the Netherlands as a domain-specific approach to metadata harmonization. It integrates both the Dutch Application Profile of the European DCAT-AP (DCAT-AP NL) [17] and HealthDCAT-AP, thereby offering a structured representation for consistently describing and organizing health-related datasets and catalogs.

### Current Metadata-Provisioning Efforts

Building on this foundation of existing standards, the FDP specification [18] provides a standardized, layered architecture for exposing metadata in a FAIR-compliant, machine-actionable manner. An FDP [19] is a metadata service designed to support the publication and discovery of metadata according to the FAIR principles. It provides a Representational State Transfer API for the creation, storage, and retrieval of FAIR-compliant metadata. Serving a dual role enables data owners to expose metadata about their digital objects in a FAIR manner and at the same time facilitates metadata discovery by consumers seeking to access and use the resources.

Metadata catalogs for datasets play a critical role in enabling FAIR data principles [2]. Several well-established national and international metadata cataloging efforts exist, most of which leverage the aforementioned W3C standards to represent the metadata about datasets that are well structured and machine-readable. The following support the representation of metadata for health-related datasets:

- Health-RI Metadata Catalogue [20]: maintained by the Health-RI initiative, provides a comprehensive overview of health and life sciences research data, offering metadata that describe available datasets and resources across diverse domains, including electronic records, images, biomaterials, omics data, and collections. The catalog’s metadata schema (Health-RI metadata schema) is based on DCAT-AP and DCAT-AP NL and extends it with health-data related elements from the HealthDCAT-AP. The catalog uses the FDP specification as part of its technical architecture for exposing and harvesting metadata. Metadata entries include essential descriptive elements such as creation date, authorship, and data access URLs, thus fostering cross-domain discoverability for secondary data reuse.
- Maelstrom Catalogue [21]: developed by Maelstrom Research in Canada, provides a centralized platform to explore metadata from population-based cohort studies and research networks worldwide. The catalog describes datasets at both the study and variable level, covering information such as study design, population characteristics, collected variables, and data collection methods. It establishes its own metadata standardization through its harmonization toolkit, combined with open-source software such as Mica (Maelstrom Research) and Opal (Maelstrom Research) [22]. This fine-grained metadata supports harmonization, cross-study comparisons, and secondary use of cohort data, thereby facilitating large-scale integrative health research. Its metadata model was designed to be compatible with existing metadata standards, including the DDI, thus allowing for improved interoperability and reuse [23].
- I14Y Interoperability Platform [24]: operated by the Swiss Federal Statistical Office, I14Y is the national platform for publishing and discovering data, electronic interfaces, and government services in Switzerland. The platform follows the DCAT and DCAT-AP CH standards, has a cross-domain scope, and is not specifically tailored to health data.
- Health Study Hub [25]: developed by NFDI4Health (National Research Data Infrastructure for Personal Health Data) in Germany, the Health Study Hub provides a platform for publishing and discovering metadata for clinical, epidemiological, and public health research. The portal is built upon open-source software while artifacts are described following the NFDI4Health Metadata Schema [26]. The schema adapts elements from established schemas, such as DataCite [27], ClinicalTrials.gov [28], or Maelstrom, and is implemented as Fast Healthcare Interoperability Resources (FHIR) [29] profiles. This provides the structural backbone for registering and harmonizing metadata from diverse study types, enabling semantic interoperability, variable-level search, and machine-actionable access to study information without exposing sensitive data [26,30]. The NFDI4Health Metadata Schema also has planned mapping to HealthDCAT-AP to prepare the Health Study Hub services for interoperability with the European Health Data Space [26].

The examples demonstrate that metadata catalogs improve dataset visibility and accessibility. However, much existing catalogs' metadata is not tailored to health-related datasets and remains limited to top-level dataset descriptions, providing little insight into the actual structure or semantics of the underlying data. A comparison of the characteristics of existing metadata catalogs is provided in Table 1.

**Table 1.** Comparison of existing metadata catalogs, based on criteria such as the use of metadata standards, availability of Findable, Accessible, Interoperable, and Reusable (FAIR) data infrastructure, scope of technical implementation, and richness of available metadata.

	Health-RI^a^	Maelstrom	I14Y Interoperability Platform	Health Study Hub
Metadata standards	DCAT^b^-AP^c^, DCAT-AP NL^d^, HealthDCAT-AP^e^	Custom domain-specific model	DCAT-AP, DCAT-AP CH^f^	HL7^g^ FHIR-based^h^ model, supports export of metadata to Dublin Core Terms and Schema.org
FDP^i^ infrastructure	Yes	No	No	No
Technical implementation	CKAN^j^ Portal, FDP frontend and backend	Opal and Mica	Dedicated frontend and backend	Opal and Mica, dedicated frontend and backend
Quantitative metadata	Basic counts, for example, number of patients and population coverage	Study-level counts and cohort size indicators	Basic counts, for example, number of individuals	Study-level counts and cohort size indicators
Interactive visualization	No	Dashboard for cohorts	Visualization of concept and structure	Dashboard for cohorts
Machine-readable API	Yes	Possible for local implementation	Yes	Yes

a Health-RI: Health Research Infrastructure.

b DCAT: Data Catalog Vocabulary.

c DCAT-AP: DCAT Application Profile for data portals in Europe.

d DCAT-AP NL: Dutch Application Profile of the European DCAT-AP.

e HealthDCAT-AP: HealthDCAT Application Profile.

f DCAT-AP CH: DCAT Application Profile for data portals in Switzerland.

g HL7: Health Level 7.

h FHIR: Fast Healthcare Interoperability Resources.

i FDP: FAIR Data Point.

j CKAN: Comprehensive Knowledge Archive Network.

### SPHN and Secondary Data Usage

In Switzerland, SPHN was launched in 2017 by the Swiss Federal Government to develop a sustainable and interoperable national data infrastructure for health-related research. SPHN promotes the sharing of clinical and omics data across institutions, supported by a semantic framework based on RDF and ontologies [31,32]. This framework consists of a set of data schemas: the SPHN RDF Schema as the foundation, along with related project-specific schemas that extend it. Together, these schemas define the semantics and constraints to which SPHN data must conform. To foster FAIR data practices within the network, SPHN encourages the publication of structured metadata describing datasets, thereby enhancing transparency and promoting the responsible reuse of data. A key enabler for effective secondary use of health data is the ability for researchers to identify, explore, and evaluate existing datasets before initiating new data collection efforts or collaborations.

To address this need, SPHN developed the SPHN Metadata Catalog [33], a national infrastructure that provides centralized access to semantically rich metadata for health-related datasets. While primarily focused on datasets generated by SPHN-funded projects, the catalog is designed to scale and support metadata from other relevant and related initiatives in Switzerland. The SPHN Metadata Catalog offers HealthDCAT-AP-compliant metadata, an FDP (including a public SPARQL Protocol and RDF Query Language [SPARQL] endpoint), aggregated instance counts of the knowledge graphs underlying the metadata (ie, quantitative metadata), as well as metadata visualization features [34]. These features allow users to inspect and interactively explore dataset content, such as variables and their relations, data types and coding systems used, and summary statistics that reflect the abundance of specific data elements without exposing sensitive or individual-level data. This bridges the gap between high-level dataset overviews and technical data dictionaries and supports informed decision-making. In this paper, we describe the design and implementation of the SPHN Metadata Catalog, including the underlying metadata model, semantic technologies, the development process, and discuss its role in enabling FAIR and scalable metadata-driven data discovery for health research in Switzerland.

## Methods

### Ethical Considerations

Ethics approval and Institutional Review Board (IRB) review were not required for this study because no identifiable patient-level information was accessed or shared.

### Metadata Collection

Systematic metadata collection is critical to ensure that research projects and their datasets are discoverable, interoperable, and reusable. To achieve this, metadata is collected along 2 complementary dimensions:

- Qualitative metadata provides descriptive and contextual information about the project, including its scope, objectives, responsible investigators, contact points, data sources, and temporal context. This information establishes the foundation for documenting the project and corresponding datasets that enable provenance and discovery.
- Quantitative metadata captures the structural information of the underlying datasets, including counts of SPHN (and project-specific) classes and properties, terminology usage, and value set usage. While derived from sensitive routine-clinical data, the aggregated outputs of this process do not contain any patient-level information or linkage between different data elements; that is, no sensitive information.

#### Qualitative Metadata

**Figure 1.**
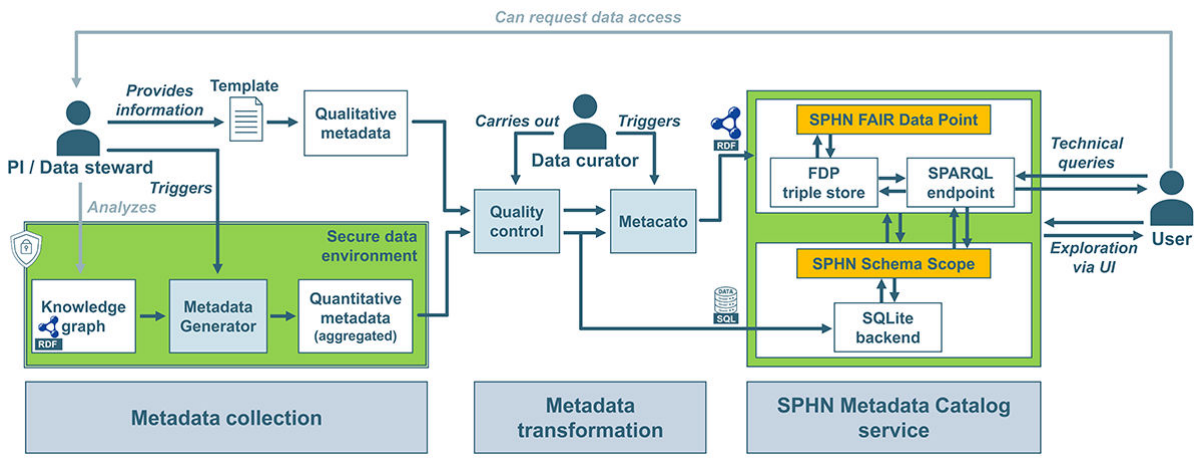
Metadata workflow: overview of collection and consumption of qualitative and quantitative metadata by the FAIR (Findable, Accessible, Interoperable, and Reusable) Data Point (FDP) and SPHN Schema Scope. FAIR: Findable, Accessible, Interoperable, and Reusable; FDP: FAIR Data Point; RDF: Resource Description Framework; SPARQL: SPARQL Protocol and RDF Query Language; SPHN: Swiss Personalized Health Network.

**Figure 2.**
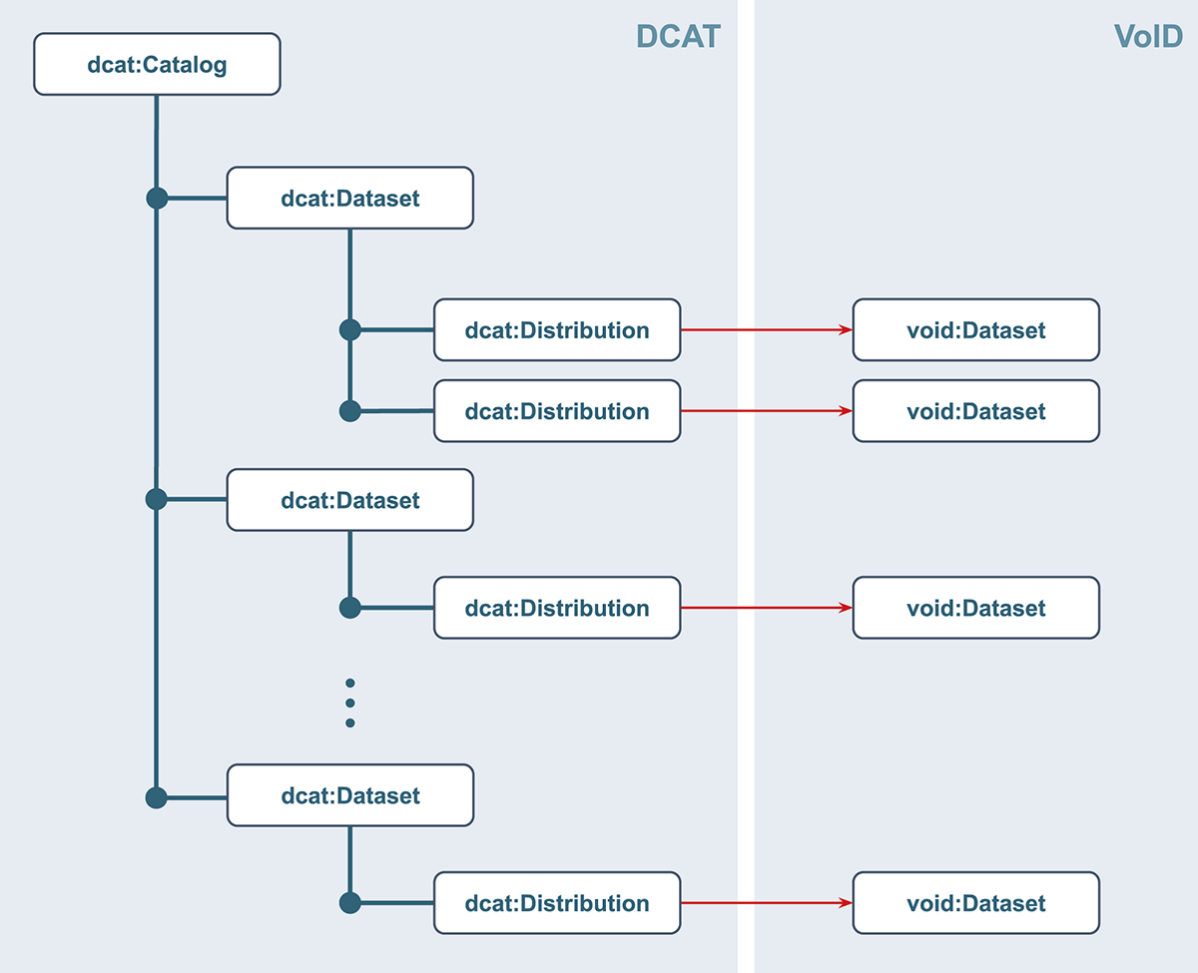
Overview of the 4 main classes in the Swiss Personalized Health Network (SPHN) Metadata Catalog Schema and their linkage. A “Catalog” can have one or more datasets, a “Dataset” can have one or more distributions, and a “Distribution” can have one or more “VoID Dataset” that further describe the distribution. DCAT: Data Catalog Vocabulary; VoID: Vocabulary of Interlinked Datasets.

**Figure 3.**
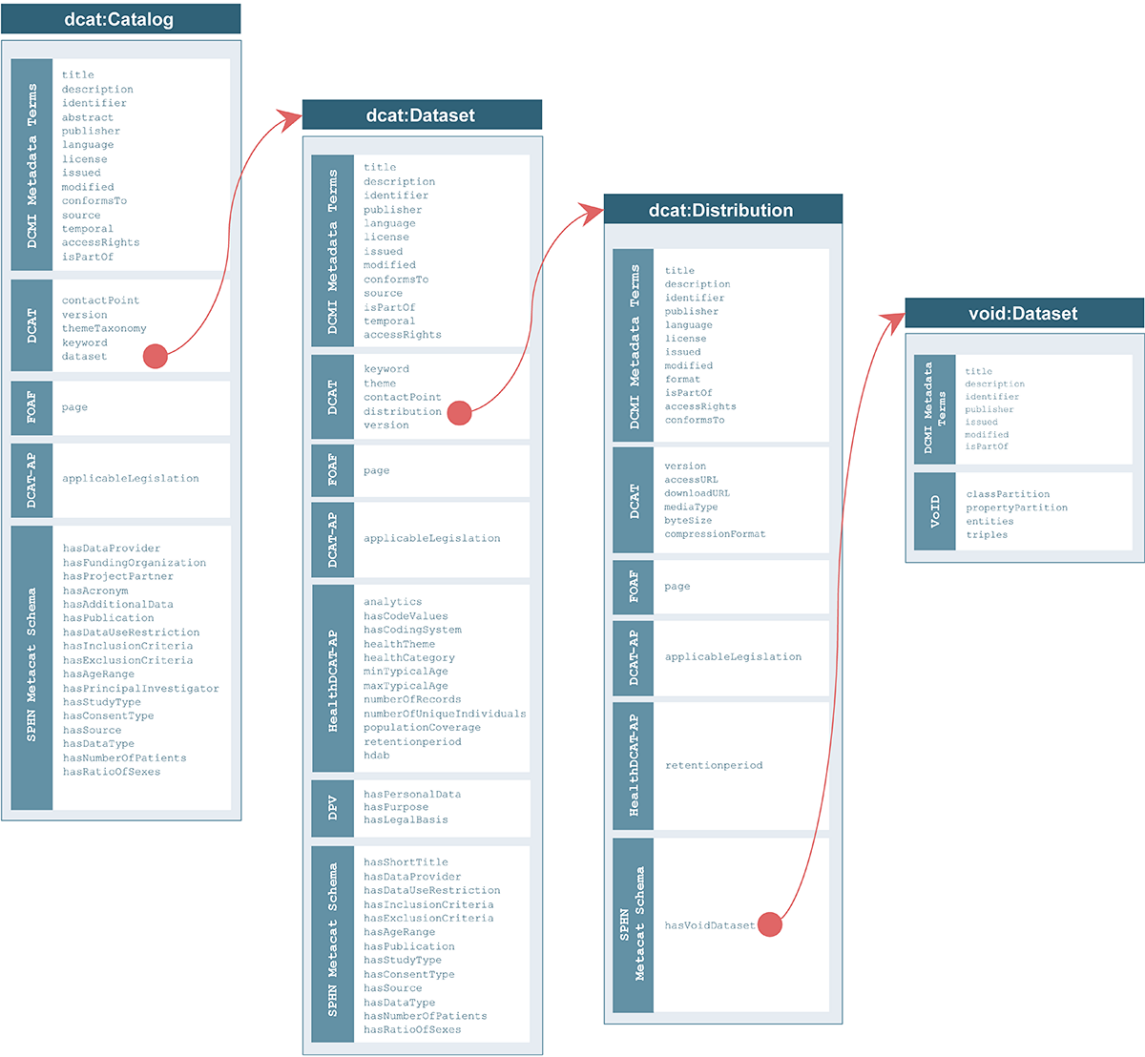
Detailed representation of the main classes of the Swiss Personalized Health Network (SPHN) Metadata Catalog Schema and their respective fields. The full content is inspectable at reference [37], DCAT: Data Catalog vocabulary; DCAT-AP: DCAT Application Profile for data portals in Europe; DCMI: Dublin Core Metadata Initiative; FOAF: Friend of a Friend vocabulary; SPHN: Swiss Personalized Health Network; DPV: Data Privacy Vocabulary; VoID: Vocabulary of Interlinked Datasets.

Qualitative metadata entails general information on a project, for example, scope and motivation, principal investigators, contact points, data sources, relevant publications, and data providers. This information is provided by the responsible person of participating projects, usually the data steward or principal investigator, using a tabular metadata template [35], licensed under Creative Commons Attribution 4.0 International License (CC BY 4.0; Figure 1). The same responsible person, when granted access to the secure data environment (eg, a trusted research environment or a hospital data warehouse), also triggers the Metadata Generator tool to extract aggregated quantitative metadata (see section “Quantitative Metadata”). The template distinguishes between metadata elements for a catalog and one or more related datasets. As a rule of thumb, a project has a catalog, and all datasets generated during the lifetime of the project can be considered individual datasets. The qualitative metadata template (Figure 1) itself is a Microsoft Excel file that data owners and data stewards can use to provide the relevant metadata. Such a template was chosen as the most accessible mode of metadata submission regardless of the level of technical expertise. The reporting of the metadata is done periodically or upon request to the projects. Metadata provided via the template to the SPHN is subsequently inspected by a data curator (Figure 1). When quality checks are passed, the curated metadata serves as the basis for creating an RDF representation by the Python-based SPHN Metadata Catalog Generator Tool (Metacato; Swiss Personalized Health Network/Swiss Institute of Bioinformatics) [36], which maps the metadata elements to terms from DCMI Metadata Terms, DCAT Version 3, DCAT-AP 3.0, and HealthDCAT-AP Release 6. The SPHN Metacato is available under an open-source GNU General Public License version 3 (GPLv3) license. In addition, certain fields are represented according to the SPHN Metadata Catalog Schema [37], a lightweight schema for documenting SPHN-specific fields. The qualitative metadata is divided into 3 components, consistent with the DCAT specification, including “Catalog”, “Dataset”, and “Distribution” where one Catalog can have one (or more) dataset, and each dataset can have one (or more) distribution (see Figure 2 for an overview and Figure 3 for details). The SPHN Metadata Catalog, with its components FDP and SPHN Schema Scope, ingests the curated metadata and makes it available to users, who can explore the metadata interactively via the user interface (UI) or via a dedicated open SPARQL query interface of the FDP for technical queries. Where suitable datasets are identified, a user can request data access to the contact specified in the metadata.

#### Quantitative Metadata

The SPHN Metadata Generator, available as a Python script or a *Dockerized container*, represents a tool that creates a count inventory of an SPHN-compliant knowledge graph of interest, yielding quantitative insights into the data density of classes and usage of terminologies. The script is openly available under an open-source GPLv3 license [38]. The SPHN Metadata Generator needs to be run within the secure data environment by an authorized person, usually a data steward or data manager.

Based on a given schema, it first generates SPARQL queries to investigate the counts of all possible traversals to each class or terminology reachable from a specific class. These queries are then run against a knowledge graph (triple store instance; eg, GraphDB [39]). Their outcomes are combined to a count inventory of the knowledge graph, which is finally made available in different formats (JavaScript object notation [JSON] and comma-separated values [CSV]) and additionally converted to an SQLite database to cater to a broad range of downstream applications. Apart from sheer count information and terminology information, no information from the knowledge graph is extracted, ensuring data protection.

In contrast to the input of the Metadata Generator (potentially sensitive data in the secure data environment), its output, that is, the quantitative metadata, contains only aggregated concept instance and terminology counts. Terminology counts represent the total count of code usages and the count of distinct codes used from a particular terminology. For clinical concepts, the metadata conveys prevalence information on a concept level, but it does not reveal any details on attributes of a particular concept. For example, it contains the number of birthdates or measurements in a dataset, as opposed to the original dataset holding clear-text birth dates or details of a measurement like time stamps, method, or its result. The aggregation also breaks all linkages between different data elements. For example, information on the presence of a birthdate and a measurement in a single original patient record is converted to independent summary counts per concept, so any connection to a particular patient is removed.

Some projects use the option to retrieve code frequencies in addition to overall terminology counts. This information is represented in VoID statistics as high-level categories (eg, top-level hierarchies for SNOMED CT [Systematized Nomenclature of Medicine Clinical Terms]) in the FDP for accessibility and as individual codes via the SPARQL endpoint. In either case, code frequencies are subject to a minimum cell-size threshold (k ≥10), with smaller counts being masked. The system also does not support cross-tabulation across multiple metadata partitions (eg, combining gender and diagnosis counts), limiting the risk of reconstructing potentially sensitive subgroup counts.

As such, the aggregated metadata can be exported from the secure data environment and published in the SPHN Metadata Catalog (Figure 1).

### SPHN FDP

**Figure 4.**
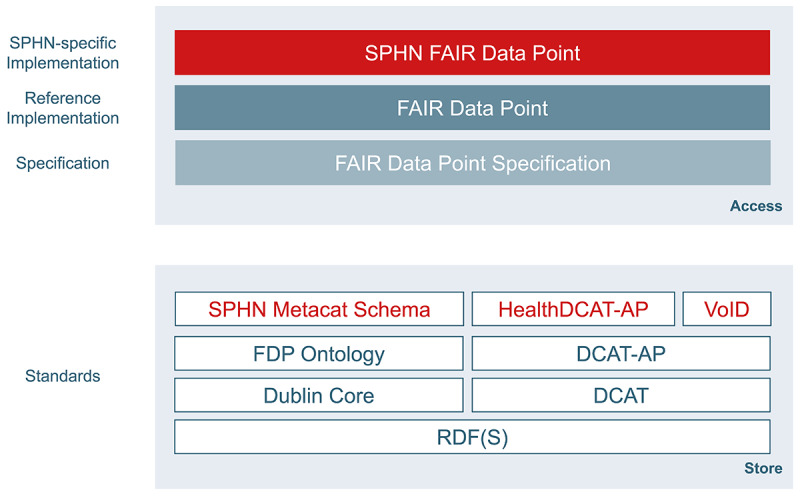
Overview of implementation of the SPHN Metadata Catalog and standards used. Red color indicates use-case-specific standards added on top of reference implementation of the FAIR Data Point (FDP). DCAT: Data Catalog Vocabulary; DCAT-AP: DCAT Application Profile for data portals in Europe; FAIR: Findable, Accessible, Interoperable, and Reusable; FDP: FAIR Data Point; RDF: Resource Description Framework; RDFS: RDF Schema; SPHN: Swiss Personalized Health Network; VoID: Vocabulary of Interlinked Datasets.

One key part of the SPHN Metadata Catalog is the SPHN FDP [33], as illustrated in Figure 4, which extends the reference implementation of the FDP specification. It ingests structured metadata (in RDF), the output of the SPHN Metacato (Figure 1). While the reference implementation of the FDP provides a standardized way to expose metadata in both human and machine-readable manner, the SPHN FDP builds on this foundation to meet specific needs of SPHN. For example, the introduction of new metadata elements that are required to describe routine clinical datasets, reuse of existing metadata elements from HealthDCAT-AP, and modification of the UI to concisely display certain metadata. By extending the reference implementation, the SPHN FDP stays aligned with the FDP specification, ensuring compatibility with broader FAIR data initiatives and tools. The SPHN FDP is backed by a GraphDB triple store as the primary store for the metadata records.

### SPHN Metadata Catalog Schema

The use of semantic standards for representing metadata ensures that metadata managed within the SPHN FDP is interoperable, machine-readable, and machine-actionable. This is critical for enabling automated discovery and reuse of health-related datasets. Since the SPHN FDP is based on the FDP reference implementation, it adopts a set of core FDP-specific standards that establish a baseline for FAIR compliance and interoperability. The DCMI Metadata Terms provide a general foundation for describing catalogs and datasets, DCAT enables datasets to be structured to support both human and machine discovery, and FDP-O extends DCAT with additional metadata elements required for managing metadata within an FDP. Building on this foundation, the SPHN FDP incorporates domain-specific standards such as HealthDCAT-AP to address the requirements of health-related metadata in Switzerland. To further complement these established standards, we introduce the SPHN Metadata Catalog Schema [37] (Figure 2). This lightweight schema follows the overall structure of DCAT and HealthDCAT-AP while accommodating SPHN-specific metadata elements that fall outside the scope of FDP-O. It is specifically designed for metadata representation within the SPHN FDP [33] as part of the SPHN Metadata Catalog.

The SPHN Metadata Catalog Schema (licensed under CC BY 4.0) consists of 2 parts: a minimal SPHN extension to DCAT and FDP-O that defines SPHN-specific properties and their semantics, and a set of Shapes Constraint Language (SHACL) shapes [40] to document and specify the structure, cardinality, and other restrictions associated with elements of the schema (see “Metadata Validation”). The shapes allow the validation of metadata in the SPHN Metadata Catalog and ensure that the records conform to the SPHN Metadata Catalog Schema. Figure 2 provides a conceptual overview of the 4 classes that form the backbone of metadata representation in the SPHN FDP. These classes are primarily based on the DCAT vocabulary (with additional use of VoID), and they structure how projects, datasets, and their technical representations are described in the SPHN Metadata Catalog.

- DCAT Catalog: represents a curated collection of metadata that describes datasets and related resources. In SPHN, each SPHN project that produces data suitable for sharing or reuse is represented as a catalog. The catalog acts as the entry point for all datasets associated with the project. A single catalog can contain one or more datasets, depending on the scope of the project.
- DCAT Dataset: represents a collection of data that is curated, published, and made accessible for discovery and reuse. In SPHN, each dataset corresponds to a set of data made available to the community for reuse by each SPHN project.
- DCAT Distribution: describes a specific representation or access mechanism of a dataset. It can correspond to a file in a specific format or an API endpoint that permits access to the data. In SPHN, when a dataset is made available in multiple technical formats (eg, CSV, RDF, and JSON), each format is represented as a separate distribution.
- VoID Dataset: describes RDF data published and maintained by a provider, often used for providing quantitative and structural metadata about datasets. In SPHN, unlike DCAT Dataset which represents the dataset conceptually, instances of VoID Dataset are used to capture quantitative metadata about a specific distribution. This includes information such as number of triples (void:triples), count of unique entities or subjects (void:entities), counts per concept class to indicate class usage (void:classPartition), counts per property to indicate property usage (void:propertyPartition), and other structural and statistical summaries that are useful for evaluating datasets.

To illustrate with an example, a project funded under SPHN may be modeled as a DCAT Catalog. Each of the project’s datasets (eg, routine clinical data, genomic data, imaging data) is represented as an individual DCAT Dataset entity of the DCAT Catalog. For each dataset, the technical formats in which the data are available (eg, RDF, CSV, and JSON) are specified in the DCAT Distribution. As a dataset may be available in multiple formats, the DCAT Distribution allows users to choose the most suitable option. Quantitative metadata about a specific distribution is finally captured in a VoID Dataset, for example, the number of subjects, classes, or triples in a graph, complemented with statistical or structural summaries useful for describing data.

Each of the 4 classes (ie, DCAT Catalog, DCAT Dataset, DCAT Distribution, and VoID Dataset) has properties from DCMI Metadata Terms, DCAT, DCAT-AP, and HealthDCAT-AP to represent resource-specific metadata in a way that is consistent and interoperable. In addition, the SPHN Metadata Catalog Schema introduces new properties for capturing SPHN-specific metadata at each level of representation to further characterize and describe resources. A complete overview of the metadata elements defined in the schema is provided in Figure 3 and in the online documentation [37].

### Metadata Validation

Ensuring the quality and conformance of metadata records within the SPHN FDP requires a reliable validation mechanism. Validation is performed against the SHACL shapes defined as part of the SPHN Metadata Catalog Schema [41] which encode the structural and semantic constraints that each metadata record must satisfy before it can be accepted into the SPHN Metadata Catalog. The full set of SHACL shapes is available at reference [40]. The SHACL shapes capture a range of constraint types. Property cardinality rules specify which metadata elements are mandatory or optional, ensuring that records carry a minimum level of descriptive completeness. Datatype and range constraints enforce that literal values conform to expected formats. Controlled vocabulary constraints restrict certain properties to values from defined value sets or ontologies. Together, these constraints validate the requirements set out by the SPHN Metadata Catalog Schema and its upstream standards, including DCAT, DCAT-AP, and HealthDCAT-AP.

Validation is integrated into the SPHN Metacato. When a metadata record is created or updated, the record is evaluated against the applicable SHACL shapes prior to being loaded into the SPHN FDP. Records that fail validation are rejected, and the resulting SHACL validation report is used to identify and mitigate the specific constraint violations. In addition to validation at creation time, periodic revalidation of existing catalog records can be triggered to assess continued conformance as the schema evolves. When the SPHN Metadata Catalog Schema is updated, existing records can be re-evaluated against the revised SHACL shapes. This supports the long-term maintainability of the catalog and ensures that the metadata records remain aligned with current schema versions.

### Metadata Accessibility

The SPHN FDP exposes metadata through its UI, allowing users to explore descriptive information about datasets and resources in a structured way. To enhance interpretability, the metadata elements displayed in the UI are not static text. Wherever possible, following the linked data principles [42], their values are presented as hyperlinks that resolve to additional resources, either within the FDP or to external portals, vocabularies, and ontologies. For example, a dataset’s declared license might be a clickable link pointing to its formal definition, or associated publications that link out to further description about the publication itself. This design ensures that users can not only read metadata but also navigate across related resources for a comprehensive view about a catalog or dataset. Beyond text-based metadata, the SPHN FDP also provides references to interactive visualization with links directly to SPHN Schema Scope (described below) in the field “Interactive Exploration” that help users contextualize datasets and their schemas. When available, users can also explore the quantitative statistics associated with a dataset via SPHN Schema Scope to get a better sense of the composition of the dataset.

### SPHN Schema Scope

The second key component of the SPHN Metadata Catalog is the SPHN Schema Scope [34], an interactive exploration tool for SPHN(-related) project schemas (Figure 1). The SPHN Schema Scope is openly available under an open-source GPLv3 license [43]. It ingests SPHN Datasets (tabular representation of the SPHN Schema; [44]) and quality-controlled quantitative metadata in the form of an SQLite database (output of the SPHN Metadata Generator; Figure 1) to generate an interactively explorable schema graph. With its intuitive graphical user interface (GUI) and the accessible visual display of data schemas, it provides an entry point to a variety of user groups, catering to a wide range of background knowledge on graph-based data models. By following the connections in the graph, users can visually navigate classes, relationships, and hierarchies defined in the schema, making it easier to understand the semantic structure of the data. The SPHN Schema Scope is aligned and cross-linked with the SPHN FDP, allowing seamless transition and access to the SPHN Metadata Catalog.

### GUI

The GUI of the SPHN Schema Scope application is based on the Shiny Web Application Framework for R (R Foundation for Statistical Computing) [45]. It is laid out to accommodate a broad range of user expertise, suitable for a quick overview for browsing to more in-depth exploration for potential secondary users. The GUI allows setting specifics of the graph representation and various general display attributes. The settings are passed to the server part of the application as parameters for graph rendering and GUI appearance. Where applicable, a tab “FAIR Data Point” is displayed, providing links to corresponding sites of the SPHN FDP [33]. User feedback has been incorporated to add functionalities considered important by the user community.

### Schema Visualization

The graph visualization of SPHN Schema Scope is primarily based on the package *visNetwork* (Network Visualization using the “vis.js“ Library) [46]. To build the schema graph, classes, relationships, data types, and standards are first extracted from a structured tabular schema representation. For accessibility, that is, human readability, not only classes but also data types and terminologies are represented as nodes while relationships (predicates) are represented as directed edges of the schema graph. These 2 sets of nodes and edges are combined into a *visNetwork-graph* object, which is rendered by the server part of a Shiny application [45]. Graph display specifications can be adjusted through the GUI.

### Graph Subsetting

The GUI also allows the selection of a subset of concepts in the schema to accommodate for different areas of interest when exploring the graph. The depth of connections can be specified to explore elements not directly connected to the user’s selection. Likewise, the directionality can be set to assess the attributes of selected concepts or the elements referencing them. This helps to get a better understanding of the schema and the connectivity of its individual elements.

To derive the graph subset, an adjacency matrix of all nodes is derived in a first step from the set of nodes and edges previously determined (see section “Schema Visualization”). From this, a distance matrix is built using the *igraph* package (Network Analysis and Visualization) [47]. This distance matrix allows selecting the nodes in the scope of the selection and depth specified by the user. A restricted set of nodes is then used as input to derive the graph as described before.

### Data Frequency Visualization

For quantitative metadata display, the schema graph can be overlaid with count information from an SQLite database generated by the SPHN Metadata Generator (see section “Quantitative Metadata”). To this end, the Shiny application connects to this SQLite database, matches the table containing the data of interest with the corresponding data schema, and queries the database during runtime. The number of instances of the data elements is retrieved, and the count information is added as an attribute to the set of nodes and edges used for visualization. For classes, the count of distinct instances is provided. For terminologies, both the count of distinct codes and the total usage count are presented. For relationships, the number of occurrences in the data is shown. The size of nodes (classes and terminologies) is scaled according to the count information. A user can interactively set specifics of the display mode like the scaling mode via the GUI. Notably, the aggregated concept counts apply to the full graph only and do not allow any combinatorics or subcohort construction. Therefore, only the full graph can be explored when visualizing data frequencies to ensure correctness of the displayed data.

### Deployment

As components of the SPHN Metadata Catalog, both SPHN FDP [33] and SPHN Schema Scope [34] are deployed on virtual machines provided by SWITCH [48], a Swiss provider for secure cloud and networking for academic institutions, and are publicly available. The applications run inside Docker containers, ensuring portability and consistency across environments. Deployment-specific parameters such as environment variables (including database and schema specifications), storage volumes, general access modalities, port mappings, and container networking are defined in a Docker Compose YAML file that orchestrates the different services. A continuous integration/continuous deployment pipeline automates testing, building, and deployment from the GitLab-based repository to the virtual server infrastructure. Any change committed to the repository, for example, an update to the Docker Compose YAML or related configuration files, triggers a pipeline that redeploys the service. External access to the SPHN Metadata Catalog components is managed by Traefik (Traefik Labs) [49], a modern reverse proxy and load balancer tightly integrated with Docker. Traefik dynamically routes incoming internet traffic to the correct service and handles concerns such as transport layer security termination, secure routing, and traffic monitoring.

## Results

### SPHN FDP

The SPHN Metadata Catalog was developed and deployed to enhance the findability and reusability of datasets generated within the SPHN. The system is in production and publicly accessible [33], and its implementation follows the FAIR principles, with a particular emphasis on providing semantically rich, machine-readable metadata to support dataset discovery. The architecture of the SPHN Metadata Catalog adheres to the FDP reference implementation [18], ensuring compliance with established standards for describing catalogs and datasets. The metadata is organized across the 3 levels—Catalog, Dataset, and Distribution—following the DCAT vocabulary. Each dataset entry is annotated with both generic and domain-specific metadata, aligning with established metadata conventions. In addition to DCAT (Figure 4), we used VoID vocabulary to represent dataset-level statistics to further describe the structure of the datasets. For SPHN-specific needs, the SPHN Metadata Catalog Schema enriches resources with health-related metadata such as inclusion and exclusion criteria, data modalities (eg, clinical, genomic, and imaging), patient age ranges, and gender distributions (see Figure 3 for the full SPHN Metadata Catalog Schema).

Metadata collection and management follow a structured and reproducible workflow. Initially, researchers provide dataset descriptions using a standardized Excel-based metadata template, structured according to the SPHN Metadata Catalog Schema. The filled templates are then processed through the SPHN Metacato that harmonizes and converts tabular entries of metadata into corresponding RDF representations. This conversion ensures syntactic and semantic consistency across datasets contributed by different projects by enforcing conformance with the underlying schema and produces metadata records compatible with the FDP infrastructure. The RDF metadata is subsequently ingested into the SPHN FDP instance, making it discoverable through both the web interface and API endpoints.

**Figure 5.**
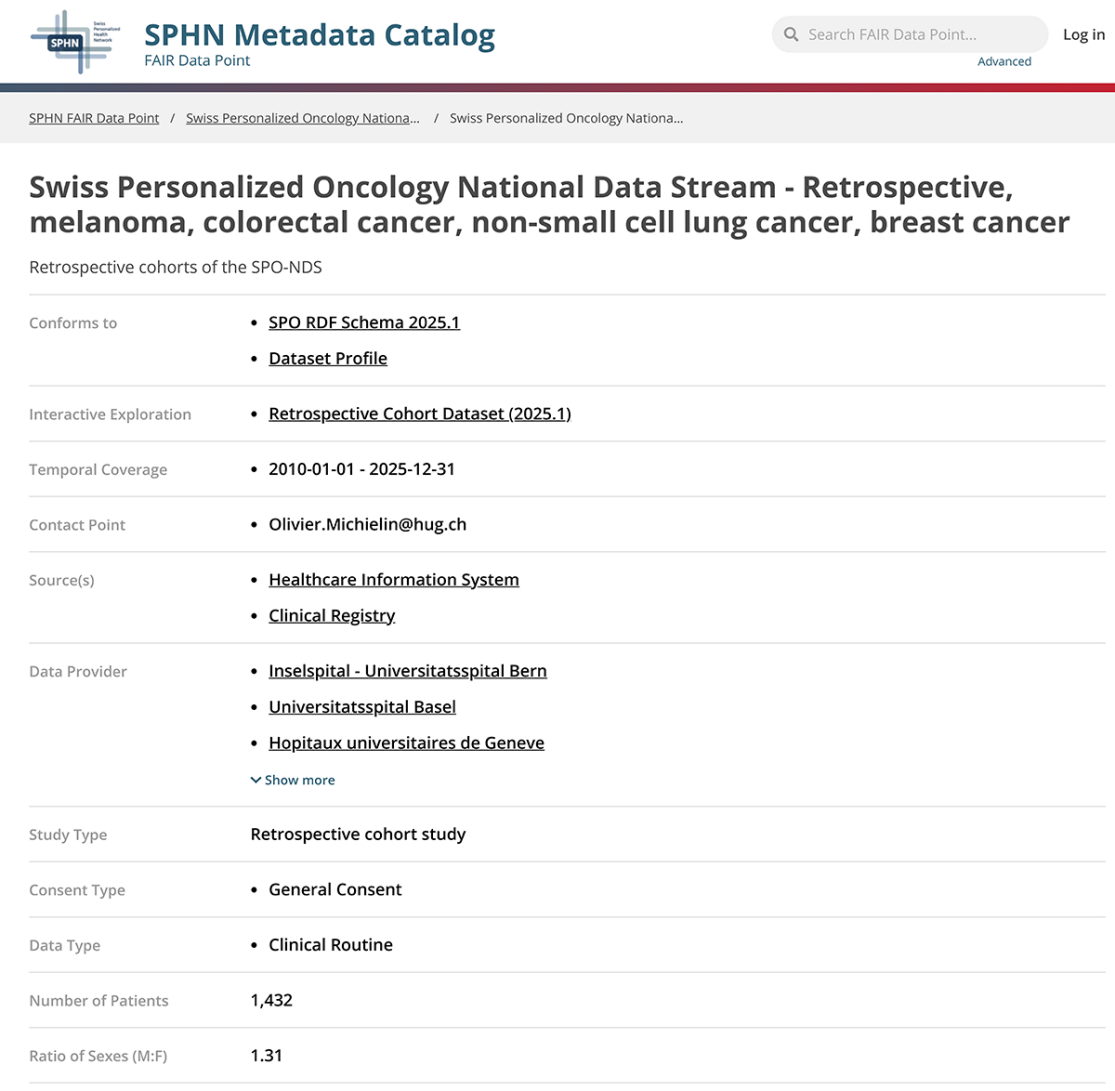
Metadata representation in the Swiss Personalized Health Network (SPHN) FAIR Data Point (FDP). Screenshot of the FDP record for the retrospective cohort dataset of the Swiss Personalized Oncology National Data Stream (SPO-NDS) [50]; FDP: FAIR Data Point; RDF: Resource Description Framework; SPHN: Swiss Personalized Health Network; SPO-NDS: Swiss Personalized Oncology National Data Stream.

To serve the needs of diverse user groups, the SPHN Metadata Catalog provides both human-readable and machine-readable views of metadata (Figure 5 and Textbox 1, respectively). Typical users can browse and search datasets through a web-based interface, while automated agents and third-party systems can access the same information programmatically using the API. This dual-access approach supports discovery and evaluation of dataset relevance without exposing sensitive patient-level data.

Textbox 1. **Resource description framework (RDF) representation of an example metadata record.**

@prefix dcterms: <http://purl.org/dc/terms/> .

@prefix dcat: <http://www.w3.org/ns/dcat#> .

@prefix foaf: <http://xmlns.com/foaf/0.1/> .

@prefix xsd: <http://www.w3.org/2001/XMLSchema#> .

@prefix ncit: <http://purl.obolibrary.org/obo/NCIT_> .

<http://fdp.dcc.sib.swiss/dataset/7dafbf3c-39f4-51b3-9e18-bfdca1ac7363> a dcat:Dataset ;

dcterms:title “Swiss Personalized Oncology National Data Stream - Retrospective, melanoma, colorectal cancer, non-small cell lung cancer, breast cancer” ;

dcterms:description “Retrospective cohorts of the SPO-NDS” ;

dcat:version “1.0” ;

dcterms:language <http://id.loc.gov/vocabulary/iso639-1/en> ;

dcterms:identifier “http://fdp.dcc.sib.swiss/dataset/7dafbf3c-39f4-51b3-9e18-bfdca1ac7363” ;

dcterms:accessRights <http://publications.europa.eu/resource/authority/access-right/RESTRICTED> ;

dcterms:publisher [

a foaf:Agent;

foaf:name “Swiss Personalized Health Network (SPHN)”

] ;

dcterms:conformsTo

<https://www.biomedit.ch/rdf/sphn-schema/spo/2025/1>,

<http://fdp.dcc.sib.swiss/profile/2f08228e-1789‐40f8-84cd-28e3288c3604> ;

dcterms:isPartOf <http://fdp.dcc.sib.swiss/catalog/88f8fde4-aff4-5c05-919c-2edba893bcd7> ;

dcterms:issued “2026-04-23T11:29:16”^^xsd:dateTime ;

dcterms:modified “2026-04-23T11:29:16”^^xsd:dateTime ;

dcterms:source

< https://www.biomedit.ch/rdf/sphn-schema/sphn/individual#HealthcareInformationSystem>,

< https://www.biomedit.ch/rdf/sphn-schema/sphn/individual#ClinicalRegistry> ;

dcterms:temporal [

a dcterms:PeriodOfTime;

dcat:endDate “2025-12-31”^^xsd:date;

dcat:startDate “2010-01-01”^^xsd:date

] ;

dcat:contactPoint <https://biomedit.ch/rdf/sphn-resource/b38990d3-0821-4032-91c8-f9a0e717aae2> ;

dcat:keyword “SPO-NDS-2025.1-Retrospective-cohort” ;

dcat:theme ncit:C84342, ncit:C16040, ncit:C17837, ncit:C20187 ;

dcat:distribution <http://fdp.dcc.sib.swiss/distribution/b3be3477-2416-5582-a573-5d67a47e76b2> ;

foaf:page <https://schemascope.dcc.sib.swiss/?project=SPO-NDS&version=2025.1&dataset=Retrospective-cohort> ;[...]

To promote discovery and reuse in biomedical research, the SPHN Metadata Catalog is indexed and linked to the FDP registry [51], thereby integrating the SPHN FDP instance into the broader FAIR ecosystem. This connection demonstrates the interoperability of the SPHN Metadata Catalog with external FAIR-aligned data infrastructures and enables federated discovery. Through this integration, datasets published within the SPHN Metadata Catalog become part of a larger network of FAIR data resources.

Textbox 1 shows an excerpt of the metadata record presented in Figure 5 in RDF (Turtle syntax). Note that the order of elements varies in the RDF serialization. The full metadata record can be found in Multimedia Appendix 1 or online in the SPHN Metadata Catalog [50].

### SPHN Schema Scope

#### Visualization of Data Schemas

To enhance transparency and facilitate understanding of the underlying data structures, the SPHN Schema Scope provides visualizations of dataset schemas. These visualizations depict concepts (classes) as nodes (boxes or circles) and predicates (relationships) as directed arrows. The platform also extracts and displays coding system usage, reporting the types of terminologies applied.

**Figure 6.**
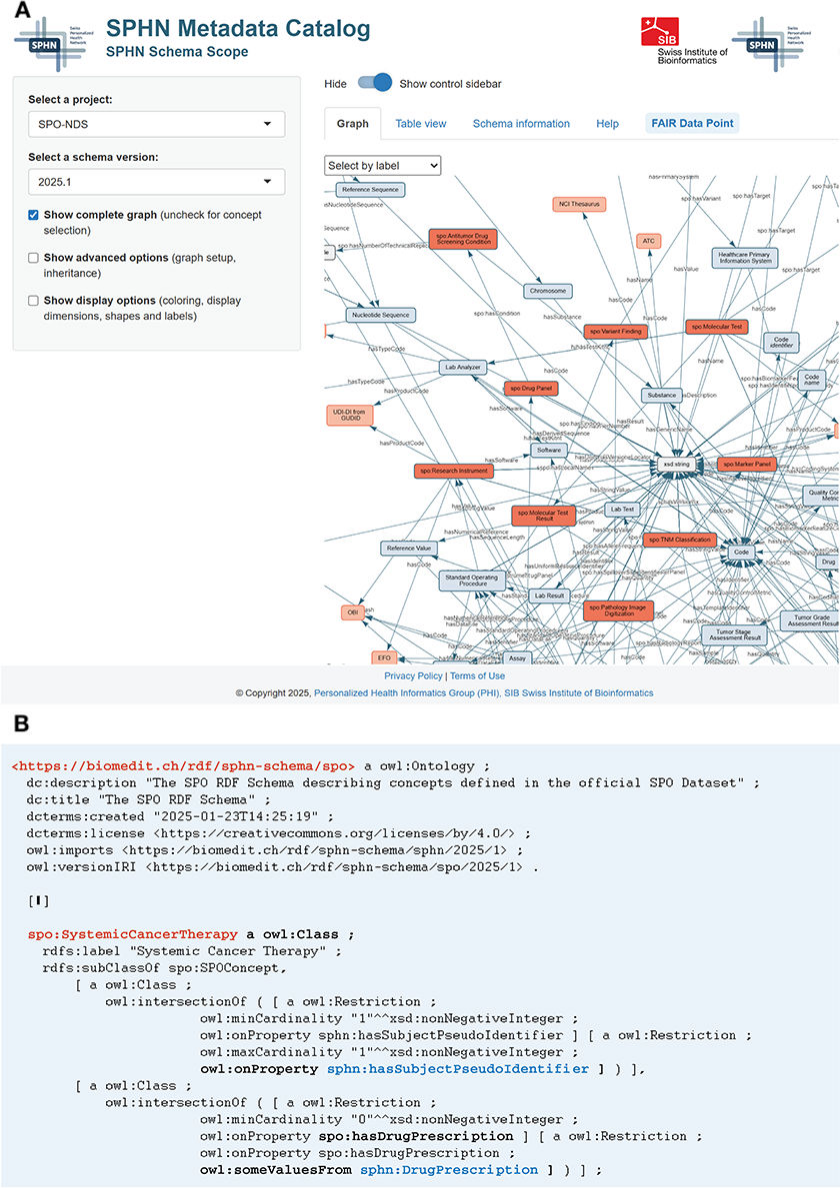
Schema visualization in the Swiss Personalized Health Network (SPHN) Schema Scope. (A) Screenshot of SPHN Schema Scope for the Swiss Personalized Oncology National Data Stream (SPO-NDS) Schema. Light blue and dark orange boxes represent concepts reused from SPHN and project-specific concepts, respectively. Terminologies are indicated in light orange. The schema can be explored interactively via reference [52]. (B) Corresponding excerpt from the SPO project-specific schema (resource description framework [RDF]-Turtle syntax). Project-specific concepts are highlighted in dark orange and reused components of the SPHN Schema in light blue. The full schema can be explored at references [53] and [54]. FDP: FAIR Data Point; RDF: Resource Description Framework; SPHN: Swiss Personalized Health Network; SPO-NDS: Swiss Personalized Oncology National Data Stream.

Schema visualizations were generated for all SPHN project-specific schemas (Figure 6A [52-54]), representing correspondences to the RDF-Turtle syntax (Figure 6B). All schemas inherit from the SPHN RDF Schema [55], ensuring a baseline interoperability across these project-specific schemas. SPHN concepts are displayed in light blue, while project-specific extensions are highlighted in dark orange, indicating customized elements. Concepts not used in a given project are omitted from the visualization to reduce complexity.

Display specifications include the selection of concepts of interest for the schema view as well as general adaptations, including setting the canvas size, displaying specific information in the property labels, graph spacing, or displaying inheritance relationships. The GUI also features a tabular view of the concept specifications, including applicable standards, cardinalities, or human-readable general and contextualized descriptions of concepts and attributes. In addition, version information on the selected schema is provided as well as license information on the standards used.

In case additional information on the selected project or dataset is available, links to corresponding resources on the SPHN FDP are provided. SPHN Schema Scope queries the SPARQL endpoint of the SPHN FDP at run-time to retrieve the stable URLs of FDP pages corresponding to the project and dataset selected in the UI.

#### Visualization of Data Frequencies in Specific Datasets

**Figure 7.**
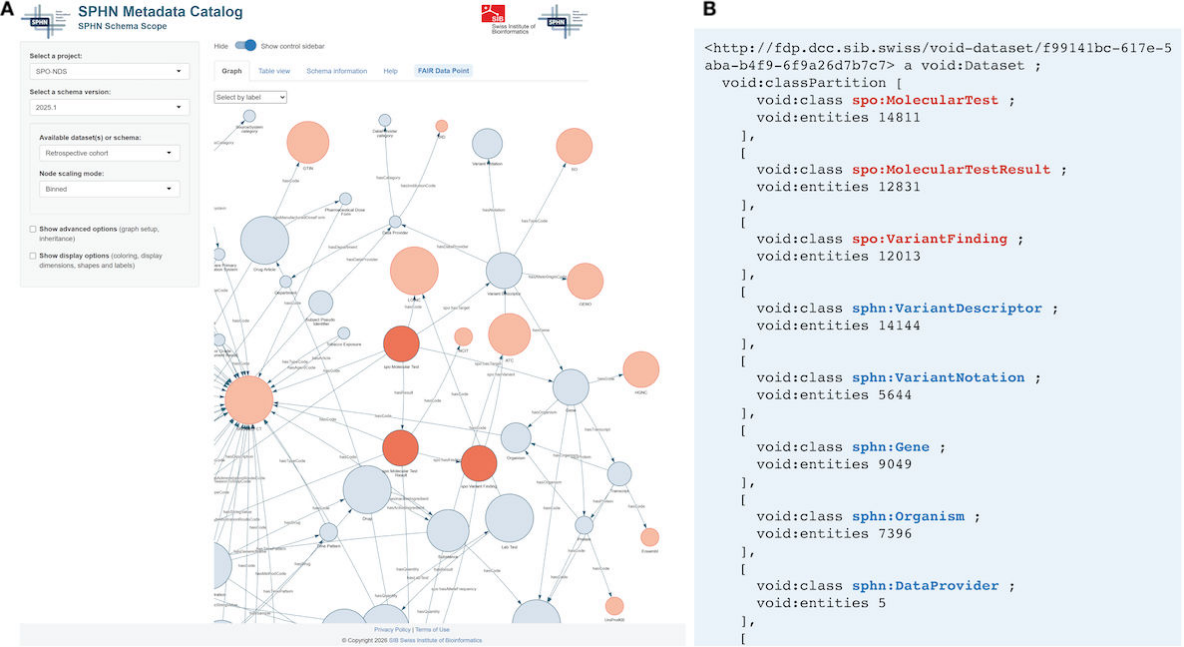
Representation of data density using quantitative metadata. (A) Screenshot of the Swiss Personalized Health Network (SPHN) Schema Scope for a project-specific dataset (Retrospective Cohort Dataset from Swiss Personalized Oncology National Data Stream [SPO-NDS]). The dataset can be explored interactively via reference [56]. The sizes of the circles correspond to the counts of the class instances (light blue: reused SPHN concepts; dark orange: project-specific concepts; light orange: terminologies). (B) Machine-readable representation of instance counts in the FAIR Data Point (FDP) using Vocabulary of Interlinked Datasets (VoID) in the distribution (Resource Description Framework [RDF]-Turtle syntax) for the dataset shown in panel (A). The full distribution record can be found online in the SPHN Metadata Catalog [57] and as Multimedia Appendix 2. FDP: FAIR Data Point; RDF: Resource Description Framework; SPHN: Swiss Personalized Health Network; SPO-NDS: Swiss Personalized Oncology National Data Stream.

For specific datasets, the SPHN Schema Scope also provides a visualization of data frequencies, offering insights into the distribution and abundance of data elements (Figure 7A) [56,57]. Dataset statistics and count information are generated by the SPHN Metadata Generator and subsequently displayed on the SPHN Metadata Catalog. This count inventory includes distinct counts of:

- Concepts (eg, diagnoses, procedures, and laboratory tests)
- Individual properties
- In the case of coding systems, the number of distinct codes and the total number of instances per coding system.

Each node representing a concept is annotated with its instance count, and the node size is scaled accordingly. Different display options are available for the scaling (ie, binned, linear, or logarithmic) to accommodate for different spreads of data abundance. Relevant edges display the prevalence of a property in the data.

The SPHN Schema Scope specifies the prevalence of clinical concepts in the dataset but does not encode or expose any population-level distributions. Quantitative information corresponding to the selection made in the SPHN Schema Scope UI is also available in the SPHN FDP in the VoID Dataset of the Distribution (RDF-Turtle syntax; Figure 7B and Multimedia Appendix 2).

The combination of schema view and data density offers an immediate and intuitive estimate of data sparsity or abundance for each concept and terminology. It allows potential secondary users to judge whether the data elements of interest are sufficiently represented and whether a dataset is suitable for the intended purpose.

### Adoption Metrics

The SPHN Metadata Catalog is a recent addition to the resources provided by the SPHN and has been publicly available since May 2025. Despite its novelty, multiple projects and institutions have contributed content already. Statistics of website visits confirm a regular use of the SPHN Metadata Catalog, bringing improved visibility for projects and datasets represented there. Several projects have contributed more than one dataset, and for multiple datasets, more than one distribution is available (eg, corresponding data in different formats). The high number of metadata fields gives the opportunity to provide rich information. Contributors embrace this option, reflected by a high completion rate even for optional elements. For example, catalogs and datasets feature 84.2% and 72.9% completion rates across a total of 38 and 48 metadata fields, respectively. Table 2 summarizes the adoption metrics reached since the catalog’s launch.

**Table 2.** Adoption metrics of the SPHN Metadata Catalog. Summary of catalog scope, metadata records (DCAT^j^ and VoID^b^ entities), user engagement, and metadata completeness. Unique site visits are reported as weekly median values. Field completion rate represents the proportion of populated metadata fields relative to all fields defined in the SPHN Metadata Catalog Schema.

Variable	Value^c^
Projects, n	18
Institutions, n	35
Metadata records, n	
Catalogs (dcat:Catalog)	18
Datasets (dcat:Dataset)	46
Distributions (dcat:Distribution)	63
VoID statistics (void:Dataset)	28
Unique site visits^d^ (per week), median (IQR)	
SPHN^e^ FAIR^f^ Data Point	53 (42‐60)
SPHN Schema Scope	34 (28‐41)
Field completion rate^g^, n/N, (n%)	
Mandatory fields	
Catalogs	5/5 (100)
Datasets	5/5 (100)
Distributions	3/3 (100)
VoID statistics	2/2 (100)
All fields	
Catalogs	32/38 (84.2)
Datasets	35/48 (72.9)
Distributions	11/22 (50.0)
VoID statistics	10/11 (90.9)

a DCAT: Data Catalog vocabulary.

b VoID: Vocabulary of Interlinked Datasets.

c As of May 10, 2026.

d Since February 2026; aggregated metrics derived using Matomo Analytics.

e SPHN: Swiss Personalized Health Network.

f FAIR: Findable, Accessible, Interoperable, and Reusable.

g Qualitative metadata fields, as specified by the SPHN Metadata Catalog Schema.

### Compliance With FAIR Principles

The catalog advances all aspects of the FAIR principles, as it provides semantically rich, detailed, and accessible metadata that go well beyond the high-level descriptions typically found in classical metadata catalogs. The metadata is accessible to both humans and machines, supporting interoperability and reuse, with minor limitations remaining in free-text fields such as data access conditions or inclusion/exclusion criteria. Table 3 summarizes, for each principle, how the SPHN Metadata Catalog enables their support.

**Table 3.** Findable, Accessible, Interoperable, and Reusable (FAIR) principle-compliance of the SPHN^a^ Metadata Catalog implementation.

FAIR principle	Characteristic
Findability	
F1: unique identifiers	Unique and persistent identifiers are defined for each catalog, dataset, and distribution.
F2: rich metadata	All metadata follow the SPHN Metadata Catalog Schema, which extends HealthDCAT-AP^b^ and provides multiple metadata elements, including extensive VoID^c^ statistics describing the scope of the data.
F3: linking data and metadata	Datasets are hosted in a restricted infrastructure; metadata records in the SPHN Metadata Catalog include persistent identifiers that provide the possibility of an explicit and traceable linkage between metadata and data within the restricted infrastructure.
F4: metadata searchable	Metadata is discoverable and searchable via an open SPARQL^d^ endpoint and the FDP^e^ interface. Metadata is available for interactive exploration via the SPHN Schema Scope interface.
Accessibility	
A1: communications protocol	Metadata is accessible via HTTPS on the SPHN Metadata Catalog.
A2: long-term availability	The SPHN Metadata Catalog is part of the national data infrastructures of the SPHN. Metadata is harvested from the SPHN Metadata Catalog into the independent national I14Y Interoperability Platform.
Interoperability	
I1: knowledge representation	Metadata is represented using Semantic Web technologies (RDF^f^, OWL^g^, SHACL^h^).
I2: controlled vocabularies	Metadata complies with DCAT^i^, the FDP Ontology, HealthDCAT-AP, and VoID.
I3: linked data	When available, direct linkage is made to health standard terminology codes (SNOMED CT^j^, LOINC^k^, ATC,^l^ etc).
Reusability	
R1: provenance and license	The SPHN Metadata Catalog Schema is available under CC-BY license. The SPHN FDP and SPHN Schema Scope are available under GNU GPL^m^ v3.0 license. Provenance metadata are available, including “Contact Point,” “Data Provider,” “Source.” Metadata complies with the FDP Ontology, DCAT, and HealthDCAT-AP, ensuring alignment with health data community standards.

a SPHN: Swiss Personalized Health Network.

b HealthDCAT-AP: Health Data Catalog Vocabulary-Application Profile.

c VoID: Vocabulary of Interlinked Datasets.

d SPARQL: SPARQL Protocol and RDF Query Language.

e FDP: FAIR Data Point.

f RDF: Resource Description Framework.

g OWL: Web Ontology Language.

h SHACL: Shapes Constraint Language.

i DCAT: Data Catalog Vocabulary.

j SNOMED CT: Systematized Nomenclature of Medicine-Clinical Terms.

k LOINC: Logical Observation Identifiers Names and Codes.

l ATC: Anatomical Therapeutic Chemical classification.

m GPL: General Public License.

## Discussion

### Overview

This paper presents the SPHN Metadata Catalog, a system designed to enhance the FAIRness of biomedical datasets within the SPHN. By providing structured, machine-readable metadata and interactive visualization, the catalog addresses key challenges in dataset discoverability, interoperability, and reusability while maintaining data privacy.

### Health Data–Specific Challenges

Data repositories like the European Genome-Phenome Archive (EGA) [58] or general-purpose open research repositories like Zenodo [59] can provide insights into the hosted content directly. In contrast, such direct provision of data is not an option for clinical or health-related data due to its potentially sensitive nature and the need to protect patient privacy. The SPHN Metadata Catalog addresses this challenge by providing researchers with rich descriptive metadata as well as information on dataset structures, data types, coding systems, and aggregated counts without revealing sensitive data. This allows insights into the data and supports secondary analyses. It provides its users with a solid information basis to decide whether datasets identified are suitable for the intended purpose. This stimulates new collaborations by making dataset characteristics transparent and accessible without exposing critical information. Users can reach out to the contact specified in the metadata to request access to data identified as fit for purpose (Figure 1).

### Data Provenance

Importantly, the catalog reflects the multilevel nature of data provenance, which is critical for auditing, verification, and reuse. Provenance is captured at the dataset level primarily through metadata describing origin, stewardship, and data sources and other characteristics, including data provider, responsible organization, source information (eg, Clinical Data Platforms, Biobanks, or OMICS), creation and update dates, versioning, and links to related resources. This enables users to quickly understand the context and scope of the dataset and, at the same time, assess its origin and stewardship even before requesting access.

For datasets that follow the SPHN RDF Schema, additional provenance can also be represented at the level of individual data elements via attributes such as source system and semantic mapping [60]. Because not all datasets in the catalog conform to the SPHN RDF Schema, the granularity of provenance information may vary across resources. In such cases, provenance is primarily represented as dataset-level metadata. This flexible approach supports transparency while accommodating dataset heterogeneity.

### Challenges With Metadata Collection

Some adoption barriers remain, as not all researchers have sufficient resources, awareness, or incentives to share their metadata with third parties. These aspects represent general issues of metadata provision, not limitations specific to the SPHN Metadata Catalog. In the context of SPHN, projects must deliver metadata at least at the closure of the project as part of the funding regulations.

For the quantitative metadata, the SPHN Metadata Generator tremendously reduces manual effort by automating the generation of count information while ensuring consistent quality and updates. However, the set of qualitative metadata needs to be collected through manually filled templates. Combined with the need for subsequent curation and quality control by the SPHN team, this process is resource-intensive. Future work will focus on streamlining qualitative metadata collection through structured and standardized mechanisms like restriction of fields to specific value sets and automated validation, thereby supporting both the metadata providers and curation teams.

### Metadata Interoperability

The schema of the SPHN Metadata Catalog fosters metadata interoperability by building on common metadata standards like DCMI Metadata Terms, DCAT, and FDP-O while also accommodating metadata elements that are of significance to SPHN. To further strengthen interoperability and alignment with other cataloging initiatives, metadata elements are harmonized with, and can be mapped to, additional profiles and schemas such as DCAT AP, DCAT-AP CH, and the Health-RI Metadata Schema. The SPHN Metadata Catalog Schema is expected to evolve alongside changing research and clinical requirements; therefore, each schema release is versioned, documented, and maintained to ensure traceability, while backward compatibility is supported through defined transformation rules that allow previously generated metadata to remain interoperable with updated versions. This approach also accommodates the ongoing evolution of frameworks such as HealthDCAT-AP, ensuring continued alignment with emerging standards. This alignment enables the exchange and reuse of metadata across different data catalogs, research infrastructures, and data platforms while maintaining consistent semantics.

Harvesting metadata from the SPHN Metadata Catalog into existing repositories, such as the national I14Y Interoperability Platform of the Swiss Federal Office of Statistics [24], has been established. Additionally, interoperability with the currently established European Health Data Portal [61] will enable federated queries across different catalogs. Both initiatives foster broader data integration and reuse.

### Conclusion

The SPHN Metadata Catalog consists of 2 core components that jointly strengthen the discoverability and reuse of SPHN datasets: the visual exploration tool SPHN Schema Scope and the standardized FDP enabling interoperable metadata access. In addition, a SPARQL endpoint supports advanced querying capabilities. Together, these components provide both intuitive and programmatic access to structured metadata, allowing users to explore, retrieve, and interrogate information in a flexible and consistent way.

The underlying technologies support interoperability not only within SPHN but also at the national and international levels, promoting responsible data reuse and enhancing the visibility and impact of SPHN projects and beyond.

## Supplementary material

10.2196/90146 Multimedia Appendix 1 The full metadata record of the Retrospective Cohort Dataset of the Swiss Personalized Oncology National Data Stream (SPO-NDS) as seen in the SPHN Metadata Catalog.

10.2196/90146 Multimedia Appendix 2 The full metadata record containing VoID statistics associated with the Distribution of the Retrospective Cohort Dataset of the Swiss Personalized Oncology National Data Stream (SPO-NDS) as seen in the SPHN Metadata Catalog.

10.2196/90146 Multimedia Appendix 3 Detailed data and code availability.

## Abbreviations

CC BY 4.0: Creative Commons Attribution 4.0 International License
DCAT: Data Catalog Vocabulary
DCAT-AP: DCAT Application profile for data portals in Europe
DCAT-AP CH: DCAT Application Profile for data portals in Switzerland
DCAT-AP NL: Dutch Application Profile of the European DCAT-AP
DCMI: Dublin Core Metadata Initiative
DDI: data documentation initiative
EGA: European Genome-Phenome Archive
FAIR: findable, accessible, interoperable, and reusable
FDP: FAIR data point
FDP-O: FAIR Data Point Ontology
FHIR: Fast Healthcare Interoperability Resources
GPLv3: GNU General Public License version 3
GUI: graphical user interface
Health-RI: Health Research Infrastructure initiative
HealthDCAT-AP: HealthDCAT Application Profile
IRB: Institutional Review Board
NFDI4Health: National Research Data Infrastructure for Personal Health Data
RDF: Resource Description Framework
SHACL: Shapes Constraint Language
SNOMED CT: Systematized Nomenclature of Medicine Clinical Terms
SPARQL: SPARQL Protocol and RDF Query Language
SPHN: Swiss Personalized Health Network
UI: user interface
VoID: Vocabulary of Interlinked Datasets
W3C: World Wide Web Consortium
