# Supplementary material for: The Swiss Personalized Health Network Metadata Catalog: Platform for Health Data Discovery and Exploration Based on Findable, Accessible, Interoperable, and Reusable Principles
Source: JMIR Med Inform. 2026 Jul 14;14:e90146. doi: 10.2196/90146 (PMC13367950; doi:10.2196/90146)
Supplement: Multimedia Appendix 1 [file medinform-v14-e90146-s001.docx]

@prefix dcterms: <http://purl.org/dc/terms/> .

@prefix dcat: <http://www.w3.org/ns/dcat#> .

@prefix foaf: <http://xmlns.com/foaf/0.1/> .

@prefix xsd: <http://www.w3.org/2001/XMLSchema#> .

@prefix ldp: <http://www.w3.org/ns/ldp#> .

@prefix fdp-o: <https://w3id.org/fdp/fdp-o#> .

@prefix ncit: <http://purl.obolibrary.org/obo/NCIT_> .

@prefix dcatap: <http://data.europa.eu/r5r/> .

@prefix healthdcatap: <http://healthdataportal.eu/ns/health#> .

<http://fdp.dcc.sib.swiss/dataset/7dafbf3c-39f4-51b3-9e18-bfdca1ac7363> a dcat:Dataset ;

dcterms:title "Swiss Personalized Oncology National Data Stream - Retrospective, melanoma, colorectal cancer, non-small cell lung cancer, breast cancer" ;

dcterms:description "Retrospective cohorts of the SPO-NDS" ;

dcat:version "1.0" ;

dcterms:language <http://id.loc.gov/vocabulary/iso639-1/en> ;

dcterms:identifier "http://fdp.dcc.sib.swiss/dataset/7dafbf3c-39f4-51b3-9e18-bfdca1ac7363" ;

dcterms:accessRights <http://publications.europa.eu/resource/authority/access-right/RESTRICTED> ;

dcterms:publisher [

a foaf:Agent;

foaf:name "Swiss Personalized Health Network (SPHN)"

] ;

dcterms:conformsTo

<https://www.biomedit.ch/rdf/sphn-schema/spo/2025/1>,

<http://fdp.dcc.sib.swiss/profile/2f08228e-1789-40f8-84cd-28e3288c3604> ;

dcterms:isPartOf <http://fdp.dcc.sib.swiss/catalog/88f8fde4-aff4-5c05-919c-2edba893bcd7> ;

dcterms:issued "2026-04-23T11:29:16"^^xsd:dateTime ;

dcterms:modified "2026-04-23T11:29:16"^^xsd:dateTime ;

dcterms:source

<https://www.biomedit.ch/rdf/sphn-schema/sphn/individual#HealthcareInformationSystem>,

<https://www.biomedit.ch/rdf/sphn-schema/sphn/individual#ClinicalRegistry> ;

dcterms:temporal [

a dcterms:PeriodOfTime;

dcat:endDate "2025-12-31"^^xsd:date;

dcat:startDate "2010-01-01"^^xsd:date

] ;

dcat:contactPoint <https://biomedit.ch/rdf/sphn-resource/b38990d3-0821-4032-91c8-f9a0e717aae2> ;

dcat:keyword "SPO-NDS-2025.1-Retrospective-cohort" ;

dcat:theme ncit:C84342, ncit:C16040, ncit:C17837, ncit:C20187 ;

dcat:distribution <http://fdp.dcc.sib.swiss/distribution/b3be3477-2416-5582-a573-5d67a47e76b2> ;

foaf:page <https://schemascope.dcc.sib.swiss/?project=SPO-NDS&version=2025.1&dataset=Retrospective-cohort> ;

dcatap:applicableLegislation

<https://www.fedlex.admin.ch/eli/cc/2013/617/de>,

<https://www.fedlex.admin.ch/eli/cc/2013/642/de>,

<https://www.fedlex.admin.ch/eli/cc/2022/491/de>,

"Cantonal data protection laws" ;

healthdcatap:hasCodingSystem

<https://browser.ihtsdotools.org/?perspective=full&conceptId1=404684003&edition=MAIN/SNOMEDCT-CH/2025-06-07>,

<https://loinc.org>,

<https://ucum.org>,

<https://www.bfs.admin.ch/bfs/de/home/statistiken/kataloge-datenbanken.assetdetail.36016195.html>,

<https://www.bfs.admin.ch/news/de/2024-0148>,

<http://www.sequenceontology.org>,

<https://atcddd.fhi.no/atc_ddd_index>,

<https://obofoundry.org/ontology/geno.html>,

<https://www.genenames.org>,

<https://www.gs1.org/standards/id-keys/gtin>,

<https://www.uid.admin.ch>,

<https://www.bfarm.de/EN/Code-systems/Classifications/ICD/ICD-O-3/_node.html>,

<https://ncithesaurus.nci.nih.gov>,

<https://hgvs-nomenclature.org>,

<https://oncotree.mskcc.org>,

<https://www.cas.org/cas-data/cas-registry>,

<https://www.ensembl.org/index.html> ;

healthdcatap:hdab [

a foaf:Agent ;

foaf:name "The SPO Consortium" ;

foaf:mbox "Olivier.Michielin@hug.ch"

] ;

healthdcatap:healthCategory

<https://biomedit.ch/rdf/sphn-metacat/sphn/health-category/EHRD>,

<https://biomedit.ch/rdf/sphn-metacat/sphn/health-category/DFMR> ;

healthdcatap:minTypicalAge 18 ;

healthdcatap:numberOfRecords 1432 ;

healthdcatap:numberOfUniqueIndividuals 1432 ;

sphn-metacat:hasDataProvider

[

a foaf:Organization ;

rdfs:label "Inselspital - Universitatsspital Bern" ;

dcterms:identifier <https://www.uid.admin.ch/Detail.aspx?uid_id=CHE-229.707.417> ;

foaf:homepage <https://www.insel.ch>

],

[

a foaf:Organization;

rdfs:label "Universitatsspital Basel" ;

dcterms:identifier <https://www.uid.admin.ch/Detail.aspx?uid_id=CHE-115.173.213> ;

foaf:homepage <https://www.unispital-basel.ch>

],

[

a foaf:Organization ;

rdfs:label "Hopitaux universitaires de Geneve" ;

dcterms:identifier <https://www.uid.admin.ch/Detail.aspx?uid_id=CHE-108.907.884> ;

foaf:homepage <https://www.hug.ch>

],

[

a foaf:Organization ;

rdfs:label "Universitatsspital Zurich" ;

dcterms:identifier <https://www.uid.admin.ch/Detail.aspx?uid_id=CHE-108.904.325> ;

foaf:homepage <https://www.usz.ch>

],

[

a foaf:Organization ;

rdfs:label "Centre hospitalier universitaire vaudois" ;

dcterms:identifier <https://www.uid.admin.ch/Detail.aspx?uid_id=CHE-108.910.225> ;

foaf:homepage <https://www.chuv.ch>

] ;

sphn-metacat:hasDataUseRestriction "Reuse conditions are regulated in the SPO Consortium Agreement and reuse is approved by the Consortium Executive Board. Third parties can apply for reuse." ;

sphn-metacat:hasAgeRange ">= 18 years old" ;

sphn-metacat:hasInclusionCriteria "Overall criteria: age at first date (incidence datetime) of diagnosis older than 18 years, approval of general consent, treated within the university hospital, biopsy assessed by NGS, date of NGS on or after 1st of January 2015" ;

sphn-metacat:hasExclusionCriteria "Overall criteria: patients treated for multiple cancers" ;

sphn-metacat:hasConsentType "General Consent" ;

sphn-metacat:hasDataType "Clinical Routine" ;

sphn-metacat:hasStudyType "Retrospective cohort study" ;

sphn-metacat:hasNumberOfPatients 1432 ;

sphn-metacat:hasRatioOfSexes "1.31" ;

sphn-metacat:hasShortTitle "SPO-NDS Retrospective cohorts" .
