# Supplementary material for: The Swiss Personalized Health Network Metadata Catalog: Platform for Health Data Discovery and Exploration Based on Findable, Accessible, Interoperable, and Reusable Principles
Source: JMIR Med Inform. 2026 Jul 14;14:e90146. doi: 10.2196/90146 (PMC13367950; doi:10.2196/90146)
Supplement: Multimedia Appendix 2 [file medinform-v14-e90146-s002.docx]

@prefix dcterms: <http://purl.org/dc/terms/> .
@prefix dcat: <http://www.w3.org/ns/dcat#> .
@prefix foaf: <http://xmlns.com/foaf/0.1/> .
@prefix xsd: <http://www.w3.org/2001/XMLSchema#> .
@prefix ldp: <http://www.w3.org/ns/ldp#> .

<http://publications.europa.eu/resource/authority/access-right/RESTRICTED> a dcterms:RightsStatement;
 <http://www.w3.org/2000/01/rdf-schema#label> "Restricted" .

<http://fdp.dcc.sib.swiss/void-dataset/f99141bc-617e-5aba-b4f9-6f9a26d7b7c7> a <http://rdfs.org/ns/void#Dataset>;
 <http://www.w3.org/2000/01/rdf-schema#label> "VOID Dataset associated with the distribution";
 dcterms:title "VOID Dataset associated with the distribution";
 <https://w3id.org/fdp/fdp-o#metadataIssued> "2026-04-24T13:46:53.745876729Z"^^xsd:dateTime;
 <https://w3id.org/fdp/fdp-o#metadataModified> "2026-04-24T13:46:53.745876729Z"^^xsd:dateTime;
 dcterms:language <http://id.loc.gov/vocabulary/iso639-1/en>;
 <https://w3id.org/fdp/fdp-o#metadataIdentifier> <http://fdp.dcc.sib.swiss/void-dataset/f99141bc-617e-5aba-b4f9-6f9a26d7b7c7#identifier>;
 dcterms:identifier "http://fdp.dcc.sib.swiss/void-dataset/f99141bc-617e-5aba-b4f9-6f9a26d7b7c7";
 dcterms:accessRights <http://publications.europa.eu/resource/authority/access-right/RESTRICTED>;
 dcterms:publisher [ a foaf:Agent;
 foaf:name "Swiss Personalized Health Network (SPHN)"
 ];
 dcterms:isPartOf <http://fdp.dcc.sib.swiss/distribution/b3be3477-2416-5582-a573-5d67a47e76b2>;
 <http://semanticscience.org/resource/SIO_000628> <http://fdp.dcc.sib.swiss/void-dataset/f99141bc-617e-5aba-b4f9-6f9a26d7b7c7/metrics/445c0a70d1e214e545b261559e2842f4>,
 <http://fdp.dcc.sib.swiss/void-dataset/f99141bc-617e-5aba-b4f9-6f9a26d7b7c7/metrics/5d27e854a9e78eb3f663331cd47cdc13>;
 dcterms:issued "2026-04-17T15:18:07"^^xsd:dateTime;
 dcterms:modified "2026-04-17T15:18:07"^^xsd:dateTime;
 <http://rdfs.org/ns/void#classPartition> [
 <http://rdfs.org/ns/void#class> <https://biomedit.ch/rdf/sphn-schema/spo#MetastasisPresence>;
 <http://rdfs.org/ns/void#entities> 2507
 ], [
 <http://rdfs.org/ns/void#class> <https://biomedit.ch/rdf/sphn-schema/sphn#TumorGradeAssessmentResult>;
 <http://rdfs.org/ns/void#entities> 159
 ], [
 <http://rdfs.org/ns/void#class> <https://biomedit.ch/rdf/sphn-schema/sphn#BodyHeightMeasurement>;
 <http://rdfs.org/ns/void#entities> 19127
 ], [
 <http://rdfs.org/ns/void#class> <https://biomedit.ch/rdf/sphn-schema/sphn#Quantity>;
 <http://rdfs.org/ns/void#entities> 373306
 ], [
 <http://rdfs.org/ns/void#class> <https://biomedit.ch/rdf/sphn-schema/sphn#GenomicDuplication>;
 <http://rdfs.org/ns/void#entities> 82
 ], [
 <http://rdfs.org/ns/void#class> <https://biomedit.ch/rdf/sphn-schema/sphn#ReferenceRange>;
 <http://rdfs.org/ns/void#entities> 190894
 ], [
 <http://rdfs.org/ns/void#class> <https://biomedit.ch/rdf/sphn-schema/sphn#VitalStatus>;
 <http://rdfs.org/ns/void#entities> 655
 ], [
 <http://rdfs.org/ns/void#class> <https://biomedit.ch/rdf/sphn-schema/sphn#TumorStageAssessmentResult>;
 <http://rdfs.org/ns/void#entities> 2171
 ], [
 <http://rdfs.org/ns/void#class> <https://biomedit.ch/rdf/sphn-schema/spo#RadiologicalOncologyDiseaseAssessment>;
 <http://rdfs.org/ns/void#entities> 19747
 ], [
 <http://rdfs.org/ns/void#class> <https://biomedit.ch/rdf/sphn-schema/sphn#Unit>;
 <http://rdfs.org/ns/void#entities> 7485
 ], [
 <http://rdfs.org/ns/void#class> <https://biomedit.ch/rdf/sphn-schema/sphn#SingleNucleotideVariation>;
 <http://rdfs.org/ns/void#entities> 3775
 ], [
 <http://rdfs.org/ns/void#class> <https://biomedit.ch/rdf/sphn-schema/sphn#Location>;
 <http://rdfs.org/ns/void#entities> 5681
 ], [
 <http://rdfs.org/ns/void#class> <https://biomedit.ch/rdf/sphn-schema/spo#SystemicCancerTherapy>;
 <http://rdfs.org/ns/void#entities> 8441
 ], [
 <http://rdfs.org/ns/void#class> <https://biomedit.ch/rdf/sphn-schema/sphn#Department>;
 <http://rdfs.org/ns/void#entities> 1
 ], [
 <http://rdfs.org/ns/void#class> <https://biomedit.ch/rdf/sphn-schema/sphn#Organism>;
 <http://rdfs.org/ns/void#entities> 7396
 ], [
 <http://rdfs.org/ns/void#class> <https://biomedit.ch/rdf/sphn-schema/sphn#Death>;
 <http://rdfs.org/ns/void#entities> 805
 ], [
 <http://rdfs.org/ns/void#class> <https://biomedit.ch/rdf/sphn-schema/spo#TNMClassification>;
 <http://rdfs.org/ns/void#entities> 3665
 ], [
 <http://rdfs.org/ns/void#class> <https://biomedit.ch/rdf/sphn-schema/sphn#DrugArticle>;
 <http://rdfs.org/ns/void#entities> 160434
 ], [
 <http://rdfs.org/ns/void#class> <https://biomedit.ch/rdf/sphn-schema/sphn#TumorStageAssessmentEvent>;
 <http://rdfs.org/ns/void#entities> 4685
 ], [
 <http://rdfs.org/ns/void#class> <https://biomedit.ch/rdf/sphn-schema/sphn#TimePattern>;
 <http://rdfs.org/ns/void#entities> 586
 ], [
 <http://rdfs.org/ns/void#class> <https://biomedit.ch/rdf/sphn-schema/sphn#VariantDescriptor>;
 <http://rdfs.org/ns/void#entities> 14144
 ], [
 <http://rdfs.org/ns/void#class> <https://biomedit.ch/rdf/sphn-schema/sphn#LabResult>;
 <http://rdfs.org/ns/void#entities> 2002112
 ], [
 <http://rdfs.org/ns/void#class> <https://biomedit.ch/rdf/sphn-schema/sphn#NucleotideSequence>;
 <http://rdfs.org/ns/void#entities> 620
 ], [
 <http://rdfs.org/ns/void#class> <https://biomedit.ch/rdf/sphn-schema/spo#OncologyDiseaseAssessmentResult>;
 <http://rdfs.org/ns/void#entities> 19733
 ], [
 <http://rdfs.org/ns/void#class> <https://biomedit.ch/rdf/sphn-schema/sphn#Discharge>;
 <http://rdfs.org/ns/void#entities> 32773
 ], [
 <http://rdfs.org/ns/void#class> <https://biomedit.ch/rdf/sphn-schema/sphn#BodyWeightMeasurement>;
 <http://rdfs.org/ns/void#entities> 53274
 ], [
 <http://rdfs.org/ns/void#class> <https://biomedit.ch/rdf/sphn-schema/sphn#DataDetermination>;
 <http://rdfs.org/ns/void#entities> 1
 ], [
 <http://rdfs.org/ns/void#class> <https://biomedit.ch/rdf/sphn-schema/sphn#CareHandling>;
 <http://rdfs.org/ns/void#entities> 473
 ], [
 <http://rdfs.org/ns/void#class> <https://biomedit.ch/rdf/sphn-schema/spo#OncologyDiseaseAssessmentEvent>;
 <http://rdfs.org/ns/void#entities> 23074
 ], [
 <http://rdfs.org/ns/void#class> <https://biomedit.ch/rdf/sphn-schema/sphn#DataProvider>;
 <http://rdfs.org/ns/void#entities> 5
 ], [
 <http://rdfs.org/ns/void#class> <https://biomedit.ch/rdf/sphn-schema/sphn#AdministrativeSex>;
 <http://rdfs.org/ns/void#entities> 1432
 ], [
 <http://rdfs.org/ns/void#class> <https://biomedit.ch/rdf/sphn-schema/sphn#BodySite>;
 <http://rdfs.org/ns/void#entities> 804
 ], [
 <http://rdfs.org/ns/void#class> <https://biomedit.ch/rdf/sphn-schema/sphn#OncologySurgery>;
 <http://rdfs.org/ns/void#entities> 7236
 ], [
 <http://rdfs.org/ns/void#class> <https://biomedit.ch/rdf/sphn-schema/sphn#Transcript>;
 <http://rdfs.org/ns/void#entities> 2075
 ], [
 <http://rdfs.org/ns/void#class> <https://biomedit.ch/rdf/sphn-schema/sphn#GenomicDeletion>;
 <http://rdfs.org/ns/void#entities> 189
 ], [
 <http://rdfs.org/ns/void#class> <https://biomedit.ch/rdf/sphn-schema/sphn#SubjectPseudoIdentifier>;
 <http://rdfs.org/ns/void#entities> 1432
 ], [
 <http://rdfs.org/ns/void#class> <https://biomedit.ch/rdf/sphn-schema/sphn#BodyHeight>;
 <http://rdfs.org/ns/void#entities> 19089
 ], [
 <http://rdfs.org/ns/void#class> <https://biomedit.ch/rdf/sphn-schema/sphn#CopyNumberVariation>;
 <http://rdfs.org/ns/void#entities> 3084
 ], [
 <http://rdfs.org/ns/void#class> <https://biomedit.ch/rdf/sphn-schema/sphn#AdministrativeCase>;
 <http://rdfs.org/ns/void#entities> 39897
 ], [
 <http://rdfs.org/ns/void#class> <https://biomedit.ch/rdf/sphn-schema/sphn#Intent>;
 <http://rdfs.org/ns/void#entities> 230
 ], [
 <http://rdfs.org/ns/void#class> <https://biomedit.ch/rdf/sphn-schema/sphn#Sample>;
 <http://rdfs.org/ns/void#entities> 501685
 ], [
 <http://rdfs.org/ns/void#class> <https://biomedit.ch/rdf/sphn-schema/sphn#MedicalDevice>;
 <http://rdfs.org/ns/void#entities> 3
 ], [
 <http://rdfs.org/ns/void#class> <https://biomedit.ch/rdf/sphn-schema/spo#BiochemicalOncologyDiseaseAssessment>;
 <http://rdfs.org/ns/void#entities> 304
 ], [
 <http://rdfs.org/ns/void#class> <https://biomedit.ch/rdf/sphn-schema/sphn#Code>;
 <http://rdfs.org/ns/void#entities> 179798
 ], [
 <http://rdfs.org/ns/void#class> <https://biomedit.ch/rdf/sphn-schema/sphn#OncologyDiagnosis>;
 <http://rdfs.org/ns/void#entities> 2778
 ], [
 <http://rdfs.org/ns/void#class> <https://biomedit.ch/rdf/sphn-schema/sphn#DrugPrescription>;
 <http://rdfs.org/ns/void#entities> 135393
 ], [
 <http://rdfs.org/ns/void#class> <https://biomedit.ch/rdf/sphn-schema/sphn#PharmaceuticalDoseForm>;
 <http://rdfs.org/ns/void#entities> 46
 ], [
 <http://rdfs.org/ns/void#class> <https://biomedit.ch/rdf/sphn-schema/sphn#SourceSystem>;
 <http://rdfs.org/ns/void#entities> 259
 ], [
 <http://rdfs.org/ns/void#class> <https://biomedit.ch/rdf/sphn-schema/spo#VariantFinding>;
 <http://rdfs.org/ns/void#entities> 12013
 ], [
 <http://rdfs.org/ns/void#class> <https://biomedit.ch/rdf/sphn-schema/sphn#ReferenceValue>;
 <http://rdfs.org/ns/void#entities> 3
 ], [
 <http://rdfs.org/ns/void#class> <https://biomedit.ch/rdf/sphn-schema/sphn#Admission>;
 <http://rdfs.org/ns/void#entities> 39845
 ], [
 <http://rdfs.org/ns/void#class> <https://biomedit.ch/rdf/sphn-schema/sphn#Consent>;
 <http://rdfs.org/ns/void#entities> 944
 ], [
 <http://rdfs.org/ns/void#class> <https://biomedit.ch/rdf/sphn-schema/sphn#GenomicInsertion>;
 <http://rdfs.org/ns/void#entities> 33
 ], [
 <http://rdfs.org/ns/void#class> <https://biomedit.ch/rdf/sphn-schema/sphn#VariantNotation>;
 <http://rdfs.org/ns/void#entities> 5644
 ], [
 <http://rdfs.org/ns/void#class> <https://biomedit.ch/rdf/sphn-schema/sphn#TumorGradeAssessment>;
 <http://rdfs.org/ns/void#entities> 159
 ], [
 <http://rdfs.org/ns/void#class> <https://biomedit.ch/rdf/sphn-schema/sphn#GenomicDelins>;
 <http://rdfs.org/ns/void#entities> 156
 ], [
 <http://rdfs.org/ns/void#class> <https://biomedit.ch/rdf/sphn-schema/sphn#DataRelease>;
 <http://rdfs.org/ns/void#entities> 1061
 ], [
 <http://rdfs.org/ns/void#class> <https://biomedit.ch/rdf/sphn-schema/sphn#TumorGradeAssessmentEvent>;
 <http://rdfs.org/ns/void#entities> 171
 ], [
 <http://rdfs.org/ns/void#class> <https://biomedit.ch/rdf/sphn-schema/sphn#Laterality>;
 <http://rdfs.org/ns/void#entities> 10
 ], [
 <http://rdfs.org/ns/void#class> <https://biomedit.ch/rdf/sphn-schema/sphn#BirthDate>;
 <http://rdfs.org/ns/void#entities> 1271
 ], [
 <http://rdfs.org/ns/void#class> <https://biomedit.ch/rdf/sphn-schema/spo#CancerTreatmentComplex>;
 <http://rdfs.org/ns/void#entities> 3233
 ], [
 <http://rdfs.org/ns/void#class> <https://biomedit.ch/rdf/sphn-schema/sphn#HealthcarePrimaryInformationSystem>;
 <http://rdfs.org/ns/void#entities> 55
 ], [
 <http://rdfs.org/ns/void#class> <https://biomedit.ch/rdf/sphn-schema/sphn#FollowUp>;
 <http://rdfs.org/ns/void#entities> 4647
 ], [
 <http://rdfs.org/ns/void#class> <https://biomedit.ch/rdf/sphn-schema/sphn#Gene>;
 <http://rdfs.org/ns/void#entities> 9049
 ], [
 <http://rdfs.org/ns/void#class> <https://biomedit.ch/rdf/sphn-schema/spo#MolecularTest>;
 <http://rdfs.org/ns/void#entities> 14811
 ], [
 <http://rdfs.org/ns/void#class> <https://biomedit.ch/rdf/sphn-schema/sphn#TobaccoExposure>;
 <http://rdfs.org/ns/void#entities> 93
 ], [
 <http://rdfs.org/ns/void#class> <https://biomedit.ch/rdf/sphn-schema/sphn#BilledDiagnosis>;
 <http://rdfs.org/ns/void#entities> 76446
 ], [
 <http://rdfs.org/ns/void#class> <https://biomedit.ch/rdf/sphn-schema/sphn#GenomicInversion>;
 <http://rdfs.org/ns/void#entities> 4
 ], [
 <http://rdfs.org/ns/void#class> <https://biomedit.ch/rdf/sphn-schema/sphn#BilledProcedure>;
 <http://rdfs.org/ns/void#entities> 35282
 ], [
 <http://rdfs.org/ns/void#class> <https://biomedit.ch/rdf/sphn-schema/sphn#Birth>;
 <http://rdfs.org/ns/void#entities> 1581
 ], [
 <http://rdfs.org/ns/void#class> <https://biomedit.ch/rdf/sphn-schema/sphn#MedicalProcedure>;
 <http://rdfs.org/ns/void#entities> 449
 ], [
 <http://rdfs.org/ns/void#class> <https://biomedit.ch/rdf/sphn-schema/sphn#DeathDate>;
 <http://rdfs.org/ns/void#entities> 645
 ], [
 <http://rdfs.org/ns/void#class> <https://biomedit.ch/rdf/sphn-schema/sphn#Country>;
 <http://rdfs.org/ns/void#entities> 178
 ], [
 <http://rdfs.org/ns/void#class> <https://biomedit.ch/rdf/sphn-schema/sphn#BodyWeight>;
 <http://rdfs.org/ns/void#entities> 52982
 ], [
 <http://rdfs.org/ns/void#class> <https://biomedit.ch/rdf/sphn-schema/sphn#ReferenceSequence>;
 <http://rdfs.org/ns/void#entities> 5003
 ], [
 <http://rdfs.org/ns/void#class> <https://biomedit.ch/rdf/sphn-schema/sphn#Drug>;
 <http://rdfs.org/ns/void#entities> 258967
 ], [
 <http://rdfs.org/ns/void#class> <https://biomedit.ch/rdf/sphn-schema/sphn#TumorSpecimen>;
 <http://rdfs.org/ns/void#entities> 1441
 ], [
 <http://rdfs.org/ns/void#class> <https://biomedit.ch/rdf/sphn-schema/sphn#HealthcareEncounter>;
 <http://rdfs.org/ns/void#entities> 118727
 ], [
 <http://rdfs.org/ns/void#class> <https://biomedit.ch/rdf/sphn-schema/sphn#LabTestEvent>;
 <http://rdfs.org/ns/void#entities> 2188907
 ], [
 <http://rdfs.org/ns/void#class> <https://biomedit.ch/rdf/sphn-schema/sphn#Age>;
 <http://rdfs.org/ns/void#entities> 20757
 ], [
 <http://rdfs.org/ns/void#class> <https://biomedit.ch/rdf/sphn-schema/sphn#LabTest>;
 <http://rdfs.org/ns/void#entities> 2632103
 ], [
 <http://rdfs.org/ns/void#class> <https://biomedit.ch/rdf/sphn-schema/sphn#Chromosome>;
 <http://rdfs.org/ns/void#entities> 2127
 ], [
 <http://rdfs.org/ns/void#class> <https://biomedit.ch/rdf/sphn-schema/sphn#Protein>;
 <http://rdfs.org/ns/void#entities> 281
 ], [
 <http://rdfs.org/ns/void#class> <https://biomedit.ch/rdf/sphn-schema/sphn#Substance>;
 <http://rdfs.org/ns/void#entities> 40196
 ], [
 <http://rdfs.org/ns/void#class> <https://biomedit.ch/rdf/sphn-schema/sphn#TherapeuticArea>;
 <http://rdfs.org/ns/void#entities> 3232
 ], [
 <http://rdfs.org/ns/void#class> <https://biomedit.ch/rdf/sphn-schema/sphn#TumorStageAssessment>;
 <http://rdfs.org/ns/void#entities> 2815
 ], [
 <http://rdfs.org/ns/void#class> <https://biomedit.ch/rdf/sphn-schema/sphn#DrugAdministrationEvent>;
 <http://rdfs.org/ns/void#entities> 712970
 ], [
 <http://rdfs.org/ns/void#class> <https://biomedit.ch/rdf/sphn-schema/sphn#RadiotherapyProcedure>;
 <http://rdfs.org/ns/void#entities> 12288
 ], [
 <http://rdfs.org/ns/void#class> <https://biomedit.ch/rdf/sphn-schema/sphn#ChromosomalLocation>;
 <http://rdfs.org/ns/void#entities> 2127
 ], [
 <http://rdfs.org/ns/void#class> <https://biomedit.ch/rdf/sphn-schema/spo#MolecularTestResult>;
 <http://rdfs.org/ns/void#entities> 12831
 ], [
 <http://rdfs.org/ns/void#class> <https://biomedit.ch/rdf/sphn-schema/sphn#GenomicPosition>;
 <http://rdfs.org/ns/void#entities> 5003
 ];
 <http://rdfs.org/ns/void#entities> 20268170;
 <http://rdfs.org/ns/void#feature> <http://www.w3.org/ns/formats/Turtle>;
 <http://rdfs.org/ns/void#propertyPartition> [
 <http://rdfs.org/ns/void#property> <https://biomedit.ch/rdf/sphn-schema/sphn#hasMaterialTypeCode>;
 <http://rdfs.org/ns/void#triples> 457183
 ], [
 <http://rdfs.org/ns/void#property> <https://biomedit.ch/rdf/sphn-schema/spo#hasBiomarkerFeatureCode>;
 <http://rdfs.org/ns/void#triples> 664
 ], [
 <http://rdfs.org/ns/void#property> <https://biomedit.ch/rdf/sphn-schema/sphn#hasExact>;
 <http://rdfs.org/ns/void#triples> 5678
 ], [
 <http://rdfs.org/ns/void#property> <https://biomedit.ch/rdf/sphn-schema/spo#hasAssessmentDateTime>;
 <http://rdfs.org/ns/void#triples> 3665
 ], [
 <http://rdfs.org/ns/void#property> <https://biomedit.ch/rdf/sphn-schema/sphn#hasSharedIdentifier>;
 <http://rdfs.org/ns/void#triples> 181977
 ], [
 <http://rdfs.org/ns/void#property> <https://biomedit.ch/rdf/sphn-schema/sphn#hasPurpose>;
 <http://rdfs.org/ns/void#triples> 189
 ], [
 <http://rdfs.org/ns/void#property> <https://biomedit.ch/rdf/sphn-schema/sphn#hasAdministrationRouteCode>;
 <http://rdfs.org/ns/void#triples> 829791
 ], [
 <http://rdfs.org/ns/void#property> <https://biomedit.ch/rdf/sphn-schema/sphn#hasProtein>;
 <http://rdfs.org/ns/void#triples> 1704
 ], [
 <http://rdfs.org/ns/void#property> <https://biomedit.ch/rdf/sphn-schema/sphn#hasDeterminationDateTime>;
 <http://rdfs.org/ns/void#triples> 20757
 ], [
 <http://rdfs.org/ns/void#property> <https://biomedit.ch/rdf/sphn-schema/sphn#hasReferenceSequence>;
 <http://rdfs.org/ns/void#triples> 5003
 ], [
 <http://rdfs.org/ns/void#property> <https://biomedit.ch/rdf/sphn-schema/sphn#hasNumericalReference>;
 <http://rdfs.org/ns/void#triples> 857210
 ], [
 <http://rdfs.org/ns/void#property> <https://biomedit.ch/rdf/sphn-schema/spo#hasNPrefix>;
 <http://rdfs.org/ns/void#triples> 1399
 ], [
 <http://rdfs.org/ns/void#property> <https://biomedit.ch/rdf/sphn-schema/sphn#hasFirstAdministrationDateTime>;
 <http://rdfs.org/ns/void#triples> 135393
 ], [
 <http://rdfs.org/ns/void#property> <https://biomedit.ch/rdf/sphn-schema/sphn#hasReasonToStopCode>;
 <http://rdfs.org/ns/void#triples> 1961
 ], [
 <http://rdfs.org/ns/void#property> <https://biomedit.ch/rdf/sphn-schema/sphn#hasCategory>;
 <http://rdfs.org/ns/void#triples> 196
 ], [
 <http://rdfs.org/ns/void#property> <https://biomedit.ch/rdf/sphn-schema/sphn#hasYear>;
 <http://rdfs.org/ns/void#triples> 1860
 ], [
 <http://rdfs.org/ns/void#property> <https://biomedit.ch/rdf/sphn-schema/sphn#hasUnit>;
 <http://rdfs.org/ns/void#triples> 373306
 ], [
 <http://rdfs.org/ns/void#property> <https://biomedit.ch/rdf/sphn-schema/sphn#hasVersion>;
 <http://rdfs.org/ns/void#triples> 3289
 ], [
 <http://rdfs.org/ns/void#property> <https://biomedit.ch/rdf/sphn-schema/sphn#hasLabTest>;
 <http://rdfs.org/ns/void#triples> 2952965
 ], [
 <http://rdfs.org/ns/void#property> <https://biomedit.ch/rdf/sphn-schema/sphn#hasRadiationQuantity>;
 <http://rdfs.org/ns/void#triples> 12260
 ], [
 <http://rdfs.org/ns/void#property> <https://biomedit.ch/rdf/sphn-schema/sphn#hasAlternateAllele>;
 <http://rdfs.org/ns/void#triples> 3775
 ], [
 <http://rdfs.org/ns/void#property> <https://biomedit.ch/rdf/sphn-schema/sphn#hasTumorPurity>;
 <http://rdfs.org/ns/void#triples> 548
 ], [
 <http://rdfs.org/ns/void#property> <https://biomedit.ch/rdf/sphn-schema/sphn#hasAdministrativeCase>;
 <http://rdfs.org/ns/void#triples> 3774886
 ], [
 <http://rdfs.org/ns/void#property> <https://biomedit.ch/rdf/sphn-schema/spo#hasNSuffix>;
 <http://rdfs.org/ns/void#triples> 1916
 ], [
 <http://rdfs.org/ns/void#property> <https://biomedit.ch/rdf/sphn-schema/sphn#hasDataDetermination>;
 <http://rdfs.org/ns/void#triples> 33818
 ], [
 <http://rdfs.org/ns/void#property> <https://biomedit.ch/rdf/sphn-schema/sphn#hasSubjectAge>;
 <http://rdfs.org/ns/void#triples> 76581
 ], [
 <http://rdfs.org/ns/void#property> <https://biomedit.ch/rdf/sphn-schema/sphn#hasCountry>;
 <http://rdfs.org/ns/void#triples> 522
 ], [
 <http://rdfs.org/ns/void#property> <https://biomedit.ch/rdf/sphn-schema/spo#hasPrimaryOncologyDiagnosis>;
 <http://rdfs.org/ns/void#triples> 237
 ], [
 <http://rdfs.org/ns/void#property> <https://biomedit.ch/rdf/sphn-schema/sphn#hasStart>;
 <http://rdfs.org/ns/void#triples> 5003
 ], [
 <http://rdfs.org/ns/void#property> <https://biomedit.ch/rdf/sphn-schema/sphn#hasInstitutionCode>;
 <http://rdfs.org/ns/void#triples> 5
 ], [
 <http://rdfs.org/ns/void#property> <https://biomedit.ch/rdf/sphn-schema/sphn#hasDeath>;
 <http://rdfs.org/ns/void#triples> 390
 ], [
 <http://rdfs.org/ns/void#property> <https://biomedit.ch/rdf/sphn-schema/sphn#hasInactiveIngredient>;
 <http://rdfs.org/ns/void#triples> 279188
 ], [
 <http://rdfs.org/ns/void#property> <https://biomedit.ch/rdf/sphn-schema/sphn#hasTime>;
 <http://rdfs.org/ns/void#triples> 212
 ], [
 <http://rdfs.org/ns/void#property> <https://biomedit.ch/rdf/sphn-schema/sphn#hasCurrentLocation>;
 <http://rdfs.org/ns/void#triples> 86396
 ], [
 <http://rdfs.org/ns/void#property> <https://biomedit.ch/rdf/sphn-schema/sphn#hasLiteralSequence>;
 <http://rdfs.org/ns/void#triples> 594
 ], [
 <http://rdfs.org/ns/void#property> <https://biomedit.ch/rdf/sphn-schema/sphn#hasManufacturedDoseForm>;
 <http://rdfs.org/ns/void#triples> 5154
 ], [
 <http://rdfs.org/ns/void#property> <https://biomedit.ch/rdf/sphn-schema/sphn#hasResult>;
 <http://rdfs.org/ns/void#triples> 2891927
 ], [
 <http://rdfs.org/ns/void#property> <https://biomedit.ch/rdf/sphn-schema/sphn#hasEnd>;
 <http://rdfs.org/ns/void#triples> 5003
 ], [
 <http://rdfs.org/ns/void#property> <https://biomedit.ch/rdf/sphn-schema/sphn#hasFrequency>;
 <http://rdfs.org/ns/void#triples> 61208
 ], [
 <http://rdfs.org/ns/void#property> <https://biomedit.ch/rdf/sphn-schema/spo#hasDetectionDateTime>;
 <http://rdfs.org/ns/void#triples> 1896
 ], [
 <http://rdfs.org/ns/void#property> <https://biomedit.ch/rdf/sphn-schema/sphn#hasName>;
 <http://rdfs.org/ns/void#triples> 194463
 ], [
 <http://rdfs.org/ns/void#property> <https://biomedit.ch/rdf/sphn-schema/spo#hasAlleleFrequency>;
 <http://rdfs.org/ns/void#triples> 8928
 ], [
 <http://rdfs.org/ns/void#property> <https://biomedit.ch/rdf/sphn-schema/sphn#hasSpecialtyName>;
 <http://rdfs.org/ns/void#triples> 3232
 ], [
 <http://rdfs.org/ns/void#property> <https://biomedit.ch/rdf/sphn-schema/sphn#hasTherapeuticArea>;
 <http://rdfs.org/ns/void#triples> 84167
 ], [
 <http://rdfs.org/ns/void#property> <https://biomedit.ch/rdf/sphn-schema/spo#hasRelatedDiagnosis>;
 <http://rdfs.org/ns/void#triples> 2882
 ], [
 <http://rdfs.org/ns/void#property> <https://biomedit.ch/rdf/sphn-schema/sphn#hasCareHandling>;
 <http://rdfs.org/ns/void#triples> 38202
 ], [
 <http://rdfs.org/ns/void#property> <https://biomedit.ch/rdf/sphn-schema/spo#hasTPrefix>;
 <http://rdfs.org/ns/void#triples> 1356
 ], [
 <http://rdfs.org/ns/void#property> <https://biomedit.ch/rdf/sphn-schema/sphn#hasMedicalDevice>;
 <http://rdfs.org/ns/void#triples> 21506
 ], [
 <http://rdfs.org/ns/void#property> <https://biomedit.ch/rdf/sphn-schema/sphn#hasChromosome>;
 <http://rdfs.org/ns/void#triples> 2127
 ], [
 <http://rdfs.org/ns/void#property> <https://biomedit.ch/rdf/sphn-schema/sphn#hasStartCytobandCode>;
 <http://rdfs.org/ns/void#triples> 2127
 ], [
 <http://rdfs.org/ns/void#property> <https://biomedit.ch/rdf/sphn-schema/sphn#hasNotation>;
 <http://rdfs.org/ns/void#triples> 8015
 ], [
 <http://rdfs.org/ns/void#property> <https://biomedit.ch/rdf/sphn-schema/sphn#hasMethodCode>;
 <http://rdfs.org/ns/void#triples> 35493
 ], [
 <http://rdfs.org/ns/void#property> <https://biomedit.ch/rdf/sphn-schema/sphn#hasGenericName>;
 <http://rdfs.org/ns/void#triples> 39057
 ], [
 <http://rdfs.org/ns/void#property> <https://biomedit.ch/rdf/sphn-schema/sphn#hasSubjectPseudoIdentifier>;
 <http://rdfs.org/ns/void#triples> 4010930
 ], [
 <http://rdfs.org/ns/void#property> <https://biomedit.ch/rdf/sphn-schema/sphn#hasSequenceLength>;
 <http://rdfs.org/ns/void#triples> 620
 ], [
 <http://rdfs.org/ns/void#property> <https://biomedit.ch/rdf/sphn-schema/sphn#hasCode>;
 <http://rdfs.org/ns/void#triples> 3045599
 ], [
 <http://rdfs.org/ns/void#property> <https://biomedit.ch/rdf/sphn-schema/spo#hasMSuffix>;
 <http://rdfs.org/ns/void#triples> 1991
 ], [
 <http://rdfs.org/ns/void#property> <https://biomedit.ch/rdf/sphn-schema/spo#hasProgressionStatusCode>;
 <http://rdfs.org/ns/void#triples> 11
 ], [
 <http://rdfs.org/ns/void#property> <https://biomedit.ch/rdf/sphn-schema/sphn#hasRecordDateTime>;
 <http://rdfs.org/ns/void#triples> 127307
 ], [
 <http://rdfs.org/ns/void#property> <https://biomedit.ch/rdf/sphn-schema/sphn#hasRankCode>;
 <http://rdfs.org/ns/void#triples> 107160
 ], [
 <http://rdfs.org/ns/void#property> <https://biomedit.ch/rdf/sphn-schema/sphn#hasLaterality>;
 <http://rdfs.org/ns/void#triples> 405
 ], [
 <http://rdfs.org/ns/void#property> <https://biomedit.ch/rdf/sphn-schema/sphn#hasSample>;
 <http://rdfs.org/ns/void#triples> 2180298
 ], [
 <http://rdfs.org/ns/void#property> <https://biomedit.ch/rdf/sphn-schema/sphn#hasLowerLimit>;
 <http://rdfs.org/ns/void#triples> 125992
 ], [
 <http://rdfs.org/ns/void#property> <https://biomedit.ch/rdf/sphn-schema/sphn#hasIncidenceDateTime>;
 <http://rdfs.org/ns/void#triples> 2777
 ], [
 <http://rdfs.org/ns/void#property> <https://biomedit.ch/rdf/sphn-schema/sphn#hasReferenceAllele>;
 <http://rdfs.org/ns/void#triples> 3775
 ], [
 <http://rdfs.org/ns/void#property> <https://biomedit.ch/rdf/sphn-schema/spo#hasTarget>;
 <http://rdfs.org/ns/void#triples> 13137
 ], [
 <http://rdfs.org/ns/void#property> <https://biomedit.ch/rdf/sphn-schema/sphn#hasTimePattern>;
 <http://rdfs.org/ns/void#triples> 790396
 ], [
 <http://rdfs.org/ns/void#property> <https://biomedit.ch/rdf/sphn-schema/spo#hasMPrefix>;
 <http://rdfs.org/ns/void#triples> 1505
 ], [
 <http://rdfs.org/ns/void#property> <https://biomedit.ch/rdf/sphn-schema/sphn#hasActiveIngredient>;
 <http://rdfs.org/ns/void#triples> 291787
 ], [
 <http://rdfs.org/ns/void#property> <https://biomedit.ch/rdf/sphn-schema/spo#hasOncologyDiseaseAssessment>;
 <http://rdfs.org/ns/void#triples> 3
 ], [
 <http://rdfs.org/ns/void#property> <https://biomedit.ch/rdf/sphn-schema/sphn#hasOrganism>;
 <http://rdfs.org/ns/void#triples> 11125
 ], [
 <http://rdfs.org/ns/void#property> <https://biomedit.ch/rdf/sphn-schema/sphn#hasTranscript>;
 <http://rdfs.org/ns/void#triples> 2231
 ], [
 <http://rdfs.org/ns/void#property> <https://biomedit.ch/rdf/sphn-schema/sphn#hasBodySite>;
 <http://rdfs.org/ns/void#triples> 235515
 ], [
 <http://rdfs.org/ns/void#property> <https://biomedit.ch/rdf/sphn-schema/sphn#hasMonth>;
 <http://rdfs.org/ns/void#triples> 1199
 ], [
 <http://rdfs.org/ns/void#property> <https://biomedit.ch/rdf/sphn-schema/sphn#hasDuration>;
 <http://rdfs.org/ns/void#triples> 372700
 ], [
 <http://rdfs.org/ns/void#property> <https://biomedit.ch/rdf/sphn-schema/sphn#hasExtractionDateTime>;
 <http://rdfs.org/ns/void#triples> 1085
 ], [
 <http://rdfs.org/ns/void#property> <https://biomedit.ch/rdf/sphn-schema/sphn#hasReportDateTime>;
 <http://rdfs.org/ns/void#triples> 2174838
 ], [
 <http://rdfs.org/ns/void#property> <https://biomedit.ch/rdf/sphn-schema/spo#hasDrugPrescription>;
 <http://rdfs.org/ns/void#triples> 4439
 ], [
 <http://rdfs.org/ns/void#property> <https://biomedit.ch/rdf/sphn-schema/sphn#hasUpperLimit>;
 <http://rdfs.org/ns/void#triples> 180380
 ], [
 <http://rdfs.org/ns/void#property> <https://biomedit.ch/rdf/sphn-schema/sphn#hasPrimarySystem>;
 <http://rdfs.org/ns/void#triples> 191
 ], [
 <http://rdfs.org/ns/void#property> <https://biomedit.ch/rdf/sphn-schema/sphn#hasStatusCode>;
 <http://rdfs.org/ns/void#triples> 944
 ], [
 <http://rdfs.org/ns/void#property> <https://biomedit.ch/rdf/sphn-schema/spo#hasFinding>;
 <http://rdfs.org/ns/void#triples> 12140
 ], [
 <http://rdfs.org/ns/void#property> <https://biomedit.ch/rdf/sphn-schema/sphn#hasCoordinateConvention>;
 <http://rdfs.org/ns/void#triples> 5003
 ], [
 <http://rdfs.org/ns/void#property> <https://biomedit.ch/rdf/sphn-schema/sphn#hasDrug>;
 <http://rdfs.org/ns/void#triples> 848363
 ], [
 <http://rdfs.org/ns/void#property> <https://biomedit.ch/rdf/sphn-schema/sphn#hasFeatureLocation>;
 <http://rdfs.org/ns/void#triples> 3199
 ], [
 <http://rdfs.org/ns/void#property> <https://biomedit.ch/rdf/sphn-schema/sphn#hasGenomicPosition>;
 <http://rdfs.org/ns/void#triples> 3931
 ], [
 <http://rdfs.org/ns/void#property> <https://biomedit.ch/rdf/sphn-schema/sphn#hasTotalCopyNumber>;
 <http://rdfs.org/ns/void#triples> 765
 ], [
 <http://rdfs.org/ns/void#property> <https://biomedit.ch/rdf/sphn-schema/sphn#hasCodingSystemAndVersion>;
 <http://rdfs.org/ns/void#triples> 185441
 ], [
 <http://rdfs.org/ns/void#property> <https://biomedit.ch/rdf/sphn-schema/sphn#hasStartDateTime>;
 <http://rdfs.org/ns/void#triples> 967794
 ], [
 <http://rdfs.org/ns/void#property> <https://biomedit.ch/rdf/sphn-schema/sphn#hasGene>;
 <http://rdfs.org/ns/void#triples> 13359
 ], [
 <http://rdfs.org/ns/void#property> <https://biomedit.ch/rdf/sphn-schema/sphn#hasDate>;
 <http://rdfs.org/ns/void#triples> 2281
 ], [
 <http://rdfs.org/ns/void#property> <https://biomedit.ch/rdf/sphn-schema/sphn#hasTypeCode>;
 <http://rdfs.org/ns/void#triples> 20728
 ], [
 <http://rdfs.org/ns/void#property> <https://biomedit.ch/rdf/sphn-schema/sphn#hasAgentCode>;
 <http://rdfs.org/ns/void#triples> 93
 ], [
 <http://rdfs.org/ns/void#property> <https://biomedit.ch/rdf/sphn-schema/sphn#hasSourceSystem>;
 <http://rdfs.org/ns/void#triples> 4473812
 ], [
 <http://rdfs.org/ns/void#property> <https://biomedit.ch/rdf/sphn-schema/sphn#hasAdmission>;
 <http://rdfs.org/ns/void#triples> 39897
 ], [
 <http://rdfs.org/ns/void#property> <https://biomedit.ch/rdf/sphn-schema/sphn#hasAssessment>;
 <http://rdfs.org/ns/void#triples> 4856
 ], [
 <http://rdfs.org/ns/void#property> <https://biomedit.ch/rdf/sphn-schema/sphn#hasEndDateTime>;
 <http://rdfs.org/ns/void#triples> 653653
 ], [
 <http://rdfs.org/ns/void#property> <https://biomedit.ch/rdf/sphn-schema/sphn#hasFractionsNumber>;
 <http://rdfs.org/ns/void#triples> 12161
 ], [
 <http://rdfs.org/ns/void#property> <https://biomedit.ch/rdf/sphn-schema/sphn#hasOriginLocation>;
 <http://rdfs.org/ns/void#triples> 23578
 ], [
 <http://rdfs.org/ns/void#property> <https://biomedit.ch/rdf/sphn-schema/sphn#hasDischarge>;
 <http://rdfs.org/ns/void#triples> 35579
 ], [
 <http://rdfs.org/ns/void#property> <https://biomedit.ch/rdf/sphn-schema/sphn#hasArticle>;
 <http://rdfs.org/ns/void#triples> 232697
 ], [
 <http://rdfs.org/ns/void#property> <https://biomedit.ch/rdf/sphn-schema/sphn#hasDataProvider>;
 <http://rdfs.org/ns/void#triples> 16637
 ], [
 <http://rdfs.org/ns/void#property> <https://biomedit.ch/rdf/sphn-schema/sphn#hasStringValue>;
 <http://rdfs.org/ns/void#triples> 535491
 ], [
 <http://rdfs.org/ns/void#property> <https://biomedit.ch/rdf/sphn-schema/sphn#hasIdentifier>;
 <http://rdfs.org/ns/void#triples> 841996
 ], [
 <http://rdfs.org/ns/void#property> <https://biomedit.ch/rdf/sphn-schema/spo#hasTSuffix>;
 <http://rdfs.org/ns/void#triples> 1981
 ], [
 <http://rdfs.org/ns/void#property> <https://biomedit.ch/rdf/sphn-schema/sphn#hasCollectionDateTime>;
 <http://rdfs.org/ns/void#triples> 503010
 ], [
 <http://rdfs.org/ns/void#property> <https://biomedit.ch/rdf/sphn-schema/sphn#hasDateTime>;
 <http://rdfs.org/ns/void#triples> 2032461
 ], [
 <http://rdfs.org/ns/void#property> <https://biomedit.ch/rdf/sphn-schema/sphn#hasDescription>;
 <http://rdfs.org/ns/void#triples> 19581
 ], [
 <http://rdfs.org/ns/void#property> <https://biomedit.ch/rdf/sphn-schema/sphn#hasInsertedSequence>;
 <http://rdfs.org/ns/void#triples> 189
 ], [
 <http://rdfs.org/ns/void#property> <https://biomedit.ch/rdf/sphn-schema/sphn#hasTargetLocation>;
 <http://rdfs.org/ns/void#triples> 21666
 ], [
 <http://rdfs.org/ns/void#property> <https://biomedit.ch/rdf/sphn-schema/spo#hasClinicalCancerStage>;
 <http://rdfs.org/ns/void#triples> 754
 ], [
 <http://rdfs.org/ns/void#property> <https://biomedit.ch/rdf/sphn-schema/sphn#hasDepartment>;
 <http://rdfs.org/ns/void#triples> 1
 ], [
 <http://rdfs.org/ns/void#property> <https://biomedit.ch/rdf/sphn-schema/sphn#hasQuantity>;
 <http://rdfs.org/ns/void#triples> 2263205
 ], [
 <http://rdfs.org/ns/void#property> <https://biomedit.ch/rdf/sphn-schema/spo#hasVariant>;
 <http://rdfs.org/ns/void#triples> 12832
 ], [
 <http://rdfs.org/ns/void#property> <https://biomedit.ch/rdf/sphn-schema/sphn#hasEndCytobandCode>;
 <http://rdfs.org/ns/void#triples> 2127
 ], [
 <http://rdfs.org/ns/void#property> <https://biomedit.ch/rdf/sphn-schema/sphn#hasIntent>;
 <http://rdfs.org/ns/void#triples> 13064
 ], [
 <http://rdfs.org/ns/void#property> <https://biomedit.ch/rdf/sphn-schema/sphn#hasNucleotideSequence>;
 <http://rdfs.org/ns/void#triples> 86
 ], [
 <http://rdfs.org/ns/void#property> <https://biomedit.ch/rdf/sphn-schema/sphn#hasAlleleOriginCode>;
 <http://rdfs.org/ns/void#triples> 12710
 ], [
 <http://rdfs.org/ns/void#property> <https://biomedit.ch/rdf/sphn-schema/sphn#hasComparator>;
 <http://rdfs.org/ns/void#triples> 469
 ], [
 <http://rdfs.org/ns/void#property> <https://biomedit.ch/rdf/sphn-schema/spo#hasDrugAdministrationEvent>;
 <http://rdfs.org/ns/void#triples> 11645
 ], [
 <http://rdfs.org/ns/void#property> <https://biomedit.ch/rdf/sphn-schema/sphn#hasDay>;
 <http://rdfs.org/ns/void#triples> 1199
 ], [
 <http://rdfs.org/ns/void#property> <https://biomedit.ch/rdf/sphn-schema/sphn#hasLastAdministrationDateTime>;
 <http://rdfs.org/ns/void#triples> 101945
 ], [
 <http://rdfs.org/ns/void#property> <https://biomedit.ch/rdf/sphn-schema/spo#hasSystemicCancerTherapy>;
 <http://rdfs.org/ns/void#triples> 5504
 ], [
 <http://rdfs.org/ns/void#property> <https://biomedit.ch/rdf/sphn-schema/sphn#hasDeletedSequence>;
 <http://rdfs.org/ns/void#triples> 345
 ];
 <http://rdfs.org/ns/void#triples> 83470856;
 dcterms:conformsTo <http://fdp.dcc.sib.swiss/profile/74310438-7c45-4fdf-ac91-2463b8bedda0> .

<http://fdp.dcc.sib.swiss/void-dataset/f99141bc-617e-5aba-b4f9-6f9a26d7b7c7#identifier>
 a <http://purl.org/spar/datacite/Identifier>;
 dcterms:identifier "http://fdp.dcc.sib.swiss/void-dataset/f99141bc-617e-5aba-b4f9-6f9a26d7b7c7" .

<http://fdp.dcc.sib.swiss/void-dataset/f99141bc-617e-5aba-b4f9-6f9a26d7b7c7/metrics/445c0a70d1e214e545b261559e2842f4>
 <http://semanticscience.org/resource/SIO_000628> <https://www.ietf.org/rfc/rfc3986.txt>;
 <http://semanticscience.org/resource/SIO_000332> <https://www.ietf.org/rfc/rfc3986.txt> .

<http://fdp.dcc.sib.swiss/void-dataset/f99141bc-617e-5aba-b4f9-6f9a26d7b7c7/metrics/5d27e854a9e78eb3f663331cd47cdc13>
 <http://semanticscience.org/resource/SIO_000628> <https://www.wikidata.org/wiki/Q8777>;
 <http://semanticscience.org/resource/SIO_000332> <https://www.wikidata.org/wiki/Q8777> .

<http://fdp.dcc.sib.swiss/profile/74310438-7c45-4fdf-ac91-2463b8bedda0> <http://www.w3.org/2000/01/rdf-schema#label>
 "VOID Dataset Profile" .
